# A Breakthrough SIA-Based Dual Assay for Simultaneous Evaluation of Antioxidant Capacity via ABTS and FRAP Mechanisms

**DOI:** 10.1021/acs.analchem.5c05489

**Published:** 2026-02-04

**Authors:** Willmann Antonio Jiménez Morales, María del Pilar Cañizares-Macías

**Affiliations:** † Facultad de Ciencias Química, 27755 Universidad Autónoma “Benito Juárez” de Oaxaca (UABJO), Av. Universidad S/N, Cinco Señores, Oaxaca de Juárez C.P. 68120, Oaxaca, Mexico; ‡ Departamento de Química Analítica, Facultad de Química, Universidad Nacional Autónoma de México, Av. Universidad 3000, Ciudad de México C.P. 04510, Mexico

## Abstract

An innovative method, termed FRAP/ABTS-SIA, was developed to simultaneously integrate the FRAP and ABTS antioxidant assays within a single sequential injection analysis (SIA) system with spectrophotometric detection. Leveraging the kinetic differences between the assays and controlling the dispersion, a compact aspiration sequence (antioxidant–FRAP–ABTS–antioxidant-water) was optimized using a central composite design, defining a flow rate of 40 μL s^–1^ and aspiration volumes of 43, 38, 38, 43, and 100 μL, respectively. The system incorporated a helical reaction coil positioned before the detector, allowing the antioxidant–FRAP bolus to react while the ABTS–antioxidant–water sequence was aspirated into the holding coil. This configuration enhanced the FRAP signal and enabled clear separation of both analytical responses. Compared to conventional batch protocols, this strategy reduced FRAP reagent concentrations by 70% and ABTS^•+^ radical concentrations by 50%. The method delivers responses within a 2 min run, achieving a throughput of ∼30 samples h^–1^. Linearity was confirmed for both assays over the range 10–120 μmol L^–1^ Trolox, with detection limits of 0.031 μmol L^–1^ (FRAP) and 0.0047 μmol L^–1^ (ABTS). Intralaboratory precision was below 2% RSD, and recoveries ranged from 97.3 to 106.2% (FRAP) and 92.8 to 105.4% (ABTS). The method was successfully applied to complex food matrices—including coffees, wines, juices, and spices—showing correlations ≥0.99 with microplate reference assays. High-throughput, reagent savings, metrological robustness, and simplified data processing position FRAP/ABTS-SIA as an efficient and reliable tool for routine antioxidant capacity evaluation in food and biomedical applications.

## Graphical abstract

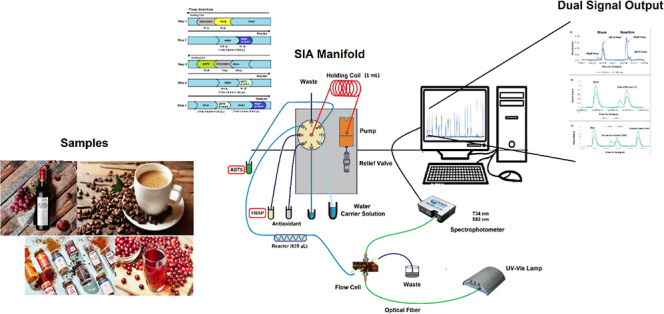

## Introduction

Oxidative stress, resulting from an imbalance between the production of reactive oxygen and nitrogen species (ROS and RNS) and the body’s capacity to neutralize them, is closely associated with the development of numerous chronic and degenerative diseases, including atherosclerosis, diabetes mellitus, chronic inflammation, neurodegenerative disorders, and certain types of cancer.^1,2^ Within this context, the accurate evaluation of antioxidant capacity in complex matrices, such as food and biological samples, is essential to understand and mitigate the effects of oxidative stress.^3,4^ Particularly in food science, antioxidants are considered substances present at low concentrations relative to an oxidizable substrate, significantly reducing or preventing adverse effects of reactive species on normal physiological functions.^5,6^ Importantly, not all reduced compounds meet this definition; only those effectively protecting biological targets from oxidation should be classified as true antioxidants.^7^ The mechanisms through which antioxidants operate are diverse and complex. These include physical barriers preventing the generation or access of ROS to sensitive biological sites, catalytic systems that divert or neutralize radicals, transition metal chelating agents preventing ROS formation, and chain-breaking antioxidants intercepting free radicals.^8,9^ These biochemical pathways primarily function through Hydrogen Atom Transfer (HAT) or Electron Transfer (ET) mechanisms to neutralize reactive species.^10,11^ Practically, these mechanisms coexist in biological and food systems, complicating the development of a single assay capable of accurately capturing all relevant antioxidant activities.^12^ The antioxidant mechanism is not fixed; it depends on chemical environment factors such as solvent type, pH, medium polarity, presence of other reducing or pro-oxidant compounds, and the nature of the radical targeted in the assay. For example, carotenoids effectively quench singlet oxygen but are inefficient against peroxyl radicals, whereas polyphenols exhibit the opposite effect.^13^ This specificity means an antioxidant may display high capacity in one assay and low capacity in another one, depending on the target radical and assay conditions.^10,14^ In response to this complexity, numerous analytical methods have been developed to assess antioxidant capacity, each based on distinct principles. Among the most used are ORAC (oxygen radical absorption capacity), ABTS (2,2′-azino-*bis*(3-ethylbenzothiazoline-6-sulfonic-acid)), DPPH (2,2′-diphenyl-1-picrylhydrazyl), and FRAP (ferric reducing antioxidant power).^15–17^ These methods vary in reactive species used, experimental conditions, sensitivity to pH and solvents, and the way results are expressed.^18^ Some techniques quantify oxidation inhibition percentages; others calculate the kinetic curve area or estimate reducing capacity through absorbance changes relative to standards like Trolox, gallic acid, or catechin.^19,20^ Method comparison is challenging due to nonstandardized radicals, reference standards, and measurement units. Additionally, certain methods measure final oxidation products, such as lipid peroxidation or fluorescent product formation, while others focus on early reaction stages, leading to different interpretations of antioxidant efficiency.^21^ For example, antioxidants like glutathione lack clear induction phases, potentially underestimating their effect in methods relying on this metric.^22^ Given that food and biological systems contain both hydrophilic and lipophilic antioxidants, selecting methods simulating real matrix conditions is crucial to avoid underestimating the total antioxidant potential.^23–25^ Moreover, methods should be distinguished between initial activity and total capacity to delay or prevent oxidation over time, a distinction frequently overlooked when these terms are used interchangeably.^26^

Part of this methodological diversity includes the FRAP and ABTS assays, widely used in food and biomedical research. The FRAP assay measures antioxidant reducing power against the ferric complex (Fe^3+^) under acidic conditions, offering simplicity and low cost. However, it does not detect hydrogen-transfer antioxidants, underestimates slow kinetics antioxidants, and is conducted at nonphysiological pH, limiting biological relevance.^10,27^ Conversely, the ABTS method assesses the neutralization of chemically generated ABTS^+•^ radical cations, applicable in both aqueous and organic media and thus suitable for hydrophilic and lipophilic antioxidants. Unlike FRAP, it operates over a wide pH range and exhibits greater versatility. Nonetheless, the ABTS^+•^ radical does not accurately represent physiological radicals, and slow reaction end points hinder standardized comparison.^28,29^ Despite these differences, both methods complement each other, allowing the evaluation of sample fractions (hydrophilic and lipophilic), different mechanisms of action (electron donation versus ferric reduction), and various physicochemical conditions. Their combined use provides a more comprehensive and robust assessment of the total antioxidant potential, especially in complex matrices like foods or natural extracts.^13,30^

In this context, sequential injection analysis (SIA) systems have become established as automated, versatile, reagent-efficient platforms facilitating antioxidant capacity determination through controlled manipulation of reagents and samples within flow systems.^31^ Several studies document successful SIA applications in antioxidant capacity assessment using ABTS,^32^ FRAP,^33^ DPPH,^34^ and ORAC methods,^35^ significantly reducing analysis times and improving precision compared to batch methods. Nevertheless, all reported SIA systems for antioxidant capacity operate sequentially, performing one determination per analytical cycle. Despite inherent advantages, no SIA systems reported thus far simultaneously integrate multiple antioxidant assays or mechanisms within a single automated procedure. Recent reviews highlight this limitation, emphasizing the need to develop analytical platforms capable of parallel evaluation of different antioxidant pathways for more comprehensive characterization.^11,26^ The concept of simultaneous determinations in flow methods refers to quantifying two or more species in a single sample using one instrumental configuration.^36,37^ However, this definition can be extended to determine the same analyte by two or more distinct methods using the same sample bolus. This approach represents a strategic alternative to quantifying an analyte through complementary reaction mechanisms, enabling result comparison and a more robust interpretation. Implementing SIA systems for simultaneous determinations in reduced sample volumes (typically tens or hundreds of microliters) presents significant challenges due to the complexity associated with adapting flow systems, as inherent dispersion phenomena of samples and reagents can compromise analytical accuracy and precision if not properly controlled.

In this work, an innovative simultaneous method for determining the antioxidant capacity was developed by integrating FRAP and ABTS assays into a single procedure. The method generated two distinct signals (peaks) corresponding to the reaction product of each assay. This was achieved through an optimized aspiration sequence, capable of controlling the dispersion of reagent volumes (ABTS and FRAP) and the sample. Both assays were conducted sequentially inline using a reactor placed before the detector. This configuration allowed the formation of two consecutive reaction product boluses, preventing mixing by adjusting the reactor length and aspirated reagent and sample volumes. As a result, continuous detection was achieved, yielding two clearly defined signals characteristic of each antioxidant assay. Notably, the proposed methodology does not require multiple detectors, unlike other flow injection systems used for simultaneous determinations. Moreover, this represents the first documented report of the simultaneous antioxidant capacity determination via SIA without coupling complementary analytical techniques. This enhances the operational simplicity and demonstrates the inherent robustness of SIA systems. Additionally, the physicochemical and hydrodynamic parameters of the FRAP/ABTS-SIA method were optimized using a central composite experimental design. Analytical quality, including linear range, limit of detection (LOD), limit of quantification (LOQ), precision, and recovery, was evaluated following international validation standards. Correlation between antioxidant capacity values obtained by the proposed method and the microplate method (for both assays) using Trolox as the standard was statistically assessed. Finally, antioxidant capacity results obtained from various food samples, including coffee, wine, juice, and spices, were compared using the proposed method and microplate method to demonstrate reliability and applicability.

## Experimental Section

### Reagents and Solutions

The reagents ABTS (2,2′-azino-*bis* (3-ethylbenzothiazoline-6-sulfonic acid)), TPTZ (2,4,6-*tri*(2-pyridyl)-*s*-triazine), K_2_S_2_O_8_, FeCl_3_·6H_2_O, HCl, and Trolox standard (6-hydroxy-2,5,7,8-tetramethylchroman-2-carboxylic acid) were employed. All reagents were of analytical grade and were purchased from Sigma-Aldrich.

Aqueous solutions of HCl at concentrations of 0.04 mol L^–1^ and 0.005 mol L^–1^, FeCl_3_·6H_2_O at 20 mmol L^–1^, and K_2_S_2_O_8_ at 2.5 mmol L^–1^ were prepared. All solutions were refrigerated at 4 °C and stored for a maximum of 15 days. The FRAP reagent or [Fe­(TPTZ)_2_]^3+^ complex were freshly prepared daily by mixing solutions of 0.005 mol L^–1^ HCl, 0.010 mol L^–1^ TPTZ (prepared in 0.04 mol L^–1^ HCl), and 0.020 mol L^–1^ FeCl_3_·6H_2_O in a volumetric ratio of 10:1:1. The ABTS^•+^ radical was generated by mixing equal volumes (1:1, v/v) of a 7 mmol L^–1^ ABTS solution with a 2.5 mmol L^–1^ K_2_S_2_O_8_ solution, followed by incubation in the dark for 12 h. This solution was stored in an amber glass container under refrigeration at 4 °C for up to 5 days. The ABTS^•+^ working solution was freshly prepared daily by diluting the stock solution in a 1:1 ratio with water. A Trolox stock solution of 400 μmol L^–1^ was prepared in distilled water and stored at 4 °C in the dark. From this stock solution, standard solutions were prepared for constructing calibration curves and for all experiments required for the development and optimization of the analytical methodology. All experiments were carried out in triplicate as an optimal balance between capturing random variability precision and maintaining efficiency in time, cost, and reagent use. ISO/IEC 17025 ^38^ mandates replicate measurements to assess repeatability and calculate measurement uncertainty, with three replicates considered the minimum for reliable estimation of within-lab precision. Also, ISO 5725 ^39^ further formalizes this by requiring at least three observations ensuring sufficient degrees of freedom for variance calculation without excessive resource use. This approach enables accurate quantification of measurement uncertainty and supports method validation under controlled conditions.

### Samples

The food matrices analyzed comprised cranberry and pomegranate juices; Carménère and Merlot red wines; coffee beans processed by honey and washed methods; green tea; and dried spices oregano, cumin, and rosemary. All samples were purchased from a local supermarket in Mexico City. To prepare the coffee, tea, cumin, oregano, and rosemary extracts, 0.9 g of each ground sample was weighed into 20 mL of distilled water preheated to 87 °C and magnetically stirred for 5 min. The suspension was subsequently filtered, the filtrate was collected, and the volume was adjusted to 25 mL in a volumetric flask. All extracts were finally diluted with distilled water.

### Instrumentation and Equipment

A FIAlab SIChrom flow-injection system was employed. The setup included a 1 mL holding loop and a 4 mL carrier-solution reservoir connected with PTFE tubing (0.70 mm i.d.). The detection module comprised two 400 μm optical fibers, a Z-flow cell (10 mm optical path length), a UV–vis-NIR light source, and an Ocean Optics USB4000 UV–vis spectrophotometer. All solutions and samples were degassed in a Branson ultrasonic bath. Sample extraction under controlled temperature and magnetic agitation was performed on a CIMAREC hot plate stirrer. Microplate antioxidant-capacity assays were conducted using a single-channel BioTek Synergy HT microplate reader equipped with a monochromator and temperature control.

Figure 1 depicts the sequential-injection analysis (SIA) system configured to evaluate antioxidant capacity simultaneously by the FRAP and ABTS assays using spectrophotometric detection (the FRAP/ABTS-SIA method). Integration of the two assays was achieved by inserting a 635 μL reactor upstream of the detector. The protocol advances through two consecutive phases: in the first, 43 μL of sample (or standard) is aspirated, followed by 38 μL of the FRAP reagent, and the mixture is immediately driven into the reactor where this first reaction proceeds; in the second, 38 μL of the ABTS^•+^ radical solution, 43 μL of the same sample (or standard), and 100 μL of distilled water are added and delivered to the same reactor to merge with the previously formed product.

**Figure 1:**
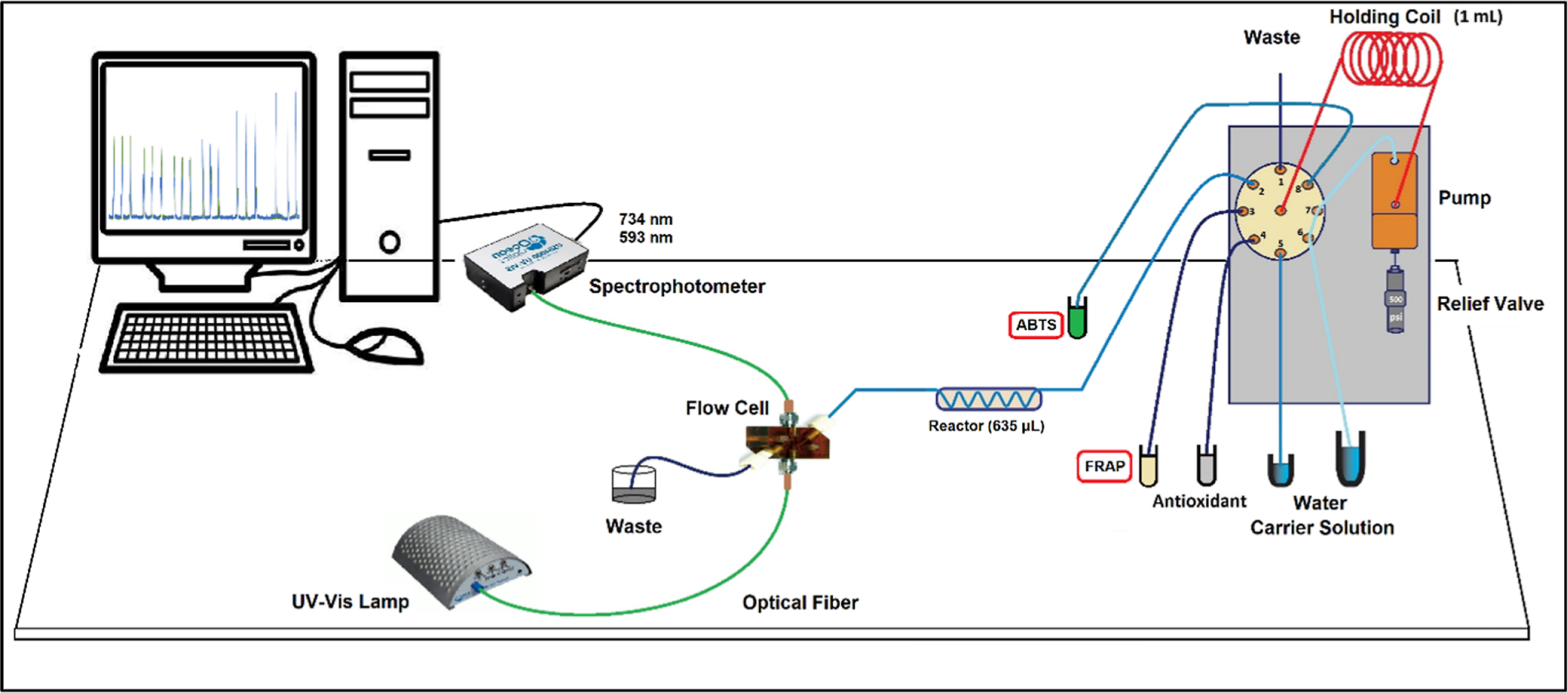
FRAP/ABTS-SIA system configuration.

The sequence summarized in Table 1 starts with filling the pump with distilled water to a total of 3 mL (step 1); the sample/standard-FRAP sequence is then introduced, and the volume is brought to 600 μL with carrier (steps 2–4); next, the ABTS^•+^ solution, a second aliquot of sample or standard, and an additional volume of 100 μL of distilled water are loaded (steps 5–7); finally, the combined contents of the reactor and holding loop are propelled to the flow cell connected to the detector set at 595 and 734 nm (step 8), yielding the spectrophotometric signals for both assays.

**Table 1:** FRAP/ABTS-SIA System Programming

step	action	port	description	flow rate (μL/s)	flow direction	time (s)
1	pump filling with carrier	6–7	the pump is filled with 3 mL of distilled water	100	reverse	30
2	antioxidant aspirate	4	43 μL of the antioxidant is aspirated into the holding coil	80	reverse	0.54
3	FRAP reagent aspirate	3	38 μL of FRAP solution is aspirated into the holding coil	80	reverse	0.48
4	dispensed to the reactor	2	600 μL is dispensed to the detector	80	forward	7.5
5	aspirated ABTS^•+^ radical	8	38 μL of ABTS solution is aspirated into the holding coil	80	reverse	0.48
6	antioxidant aspirate	4	43 μL of the antioxidant is aspirated into the holding coil	80	reverse	0.54
7	carrier aspirate	5	100 μL of water is aspirated into the holding coil	100	reverse	1
8	dispensed toward the detector^a^	2	2662 μL is dispensed from the holding coil and pump, passing through the reactor to the detector	40	forward	66.5

a Spectrophotometer set at 593 and 734 nm.

### Antioxidant Capacity Calculation

The determination of antioxidant capacity by the FRAP-SIA system was based on spectrophotometric monitoring of ferric complex [Fe (TPTZ)_2_]^3+^, whose intense absorption band diminished when reducing agents in the sample transferred electrons to Fe (III). For each injection, the initial absorbance of the reagent (*A*_0_) and the final absorbance after the reaction period (*A*_s_) were recorded. The analytical signal, *A*_FRAP_, was calculated as the difference between these two readings, as shown in eq 1:

1 $$A_{FRAP}=A_{s}-A_{0}$$

Similarly, the ABTS-SIA assay quantified the loss of color of the ABTS^•+^ radical, whose chromophore exhibits a blue-green hue. The absorbance of the radical solution was measured before (*A*_0_) and after (*A*_s_) interaction with the sample or the antioxidant standard. The corresponding signal, *A*_ABTS_, was obtained by reversing the subtraction order—because optical extinction decreases as the radical is quenched—according to eq 2:

2 $$A_{ABTS}=A_{0}-A_{s}$$

The *A*_FRAP_ and *A*_ABTS_ values served as quantitative signals during every stage of the method development and optimization. Independent calibration curves were constructed for each assay within the predefined working ranges, and the antioxidant activity of the samples was consistently expressed as μmol L^–1^ Trolox equivalents, enabling direct comparison among these determinations with prior studies that employed the same reference compound.

Trolox is widely accepted as the reference standard in antioxidant assays due to its rapid, predictable kinetics in ET-based methods (FRAP, ABTS), high aqueous solubility, chemical stability, and representative reactivity of phenolic antioxidants. Its use ensures consistent calibration curves, facilitating method comparability and validation in batch and flow systems.^10,40^

### Preliminary Optimization Studies

#### ABTS Assay Coupled to SIA

Before simultaneously coupling the ABTS assay with the FRAP assay, the ABTS protocol for individual measurement, coupled to sequential injection analysis (ABTS-SIA) as described by Lima et al. in 2005,^40^ was implemented with slight modifications to reduce the total processing time and harmonize automated antioxidant capacity determinations. Also, the aspiration volumes and antioxidant concentration intervals were implemented in alignment with the methodological framework previously established for the FRAP-SIA assay by our research group,^41^ whose optimal conditions for aspiration volumes were 33 μL for FRAP and 38 μL for antioxidant, Trolox, or sample. The Trolox standards concentration range were from 10 μmol L^–1^ to 300 μmol L^–1^.

### Preparation of the ABTS^●+^ Radical Cation

Stock solutions of ABTS (7 mmol L^–1^) and potassium peroxydisulfate (K_2_S_2_O_8_, 2.5 mmol L^–1^) were independently prepared in distilled water. Subsequently, the two reagents were mixed in a 1:1 (v/v) ratio and incubated in an amber flask protected from light at room temperature for 12 h. This incubation period, selected based on previously reported protocols, allowed for the quantitative conversion of ABTS to its radical cation (ABTS^•+^) while minimizing the risk of photodegradation.

### Configuration and Operation of the ABTS-SIA System

Following the generation of the ABTS radical cation (ABTS^•+^), the reaction mixture was diluted at ratios of 1:5 and 1:10 using 0.1 mol L^–1^ phosphate buffer (pH 7.4). The resulting solutions were subsequently introduced into the sequential injection analysis (SIA) system in accordance with the protocol detailed in Table S1 of the Supporting Information.

A preliminary evaluation of the system’s performance was conducted by injecting a Trolox standard solution (400 μmol L^–1^) as a reference antioxidant. Simultaneously, blank assays using only phosphate buffer were performed to establish baseline responses at 734 nm. The intensity of the ABTS^•+^ signal will decrease when an antioxidant reacts with it.

Quantitative assessment was achieved by injecting Trolox standards over the concentration range of 10–300 μmol L^–1^, maintaining the same injection sequence. The absorbance signals obtained were plotted and subjected to a weighted linear regression analysis. The resulting calibration curve parameters—slope, intercept, and correlation coefficient—were used to characterize the analytical response and evaluate the performance of the optimized method.

### Optimization of the FRAP/ABTS-SIA Method

Optimization focused on parameters influencing kinetic differences between FRAP and ABTS assays as well as axial dispersion in laminar flow systems. First, flow rate governs the residence time and dispersion; therefore, excessively high rates shorten the reaction time, while overly low rates increase dispersion and baseline drift. Second, aspirated volumes determine stoichiometry and zone overlap, which directly affect signal reproducibility. Moreover, the reagent concentration controls the dynamic range; insufficient or excessive levels compromise accuracy. Additionally, the aspiration sequence is essential to maintain proper zone positioning and avoid premature mixing.

Before applying the Central Composite Design (CCD), preliminary tests established aspiration order and residence time, ensuring robust optimization of hydrodynamic and chemical conditions for precise, efficient SIA performance.

### Analytical Signal Behavior without Reactor

Initially, tests to evaluate the dispersion into the system of both reactions were studied in absence working with the same manifold of Figure 1 but without the reactor coil. Although, it is well-known that the ABTS method has a faster kinetics than FRAP, our research group developed a FRAP-SIA method where the reaction was carried out in only 1.2 min,^41^ so, for this first study, four aspiration sequences were evaluated and the positions of ABTS, FRAP, and antioxidant were interchanged. These sequences involved defined volumes of ABTS^•+^, FRAP reagents, the antioxidant standard (from a 10 μmol L^–1^ Trolox standard), and water that was used with the aim of separating both signals (Table S2 of the Supporting Information).

The FRAP and ABTS^•+^ working solutions were prepared as described in the “Reagents and Solutions” section. For the ABTS assay, a 1:10 dilution of the ABTS^•+^ reagent was used exclusively. The detector wavelengths were set at 593 and 734 nm to simultaneously monitor the FRAP and ABTS responses, respectively. These wavelengths were used for all experiments.

### Reaction Coil Integration before Detector

The second study was done incorporating a helical reactor immediately before the detector to contain the bolus composed by the antioxidant standard and FRAP reagent, while the ABTS and antioxidant were aspirated (steps 5–6 of Table 1). The FRAP reagent was used undiluted to preserve its reducing capacity, whereas the ABTS^•+^ solution was diluted 1:10 with deionized water to adjust its initial absorbance within the linear range of the detector and to avoid signal saturation. For this study, Trolox standard solutions were prepared at a concentration of 140 μmol L^–1^. All measurements were corrected for their respective reaction blanks.

### Aspiration Order in the SIA Sequence

The aspiration order was optimized to maximize the sensitivity of both determinations. A one-factor-at-a-time design was applied in which the relative positions of the FRAP and ABTS^•+^ reagents were permuted. Four aspiration sequences were tested in triplicate with Trolox at 60 μmol L^–1^ (listed in Table S3 of the Supporting Information). The FRAP reagent was used at stock concentration, whereas the ABTS^•+^ solution was diluted 1:5 to position its initial absorbance within the detector’s linear range. The used aspirated volumes for these experiments were those obtained for the ABTS-SIA method, described below, and the FRAP-SIA method reported by our work group,^41^ so for ABTS and FRAP, they were 38 μL and for the antioxidant, it was 33 μL, with a a flow rate of 35 μL s^–1^. All measurements were corrected using their respective reaction blanks, and the results were expressed as absorbance.

The resulting data set served to identify the aspiration sequence that delivers the highest analytical response for the combined FRAP/ABTS-SIA method.

### Hydrodynamic and Chemical Parameters

Hydrodynamic factors such as flow rate, aspirated volumes, and reagent concentration exhibit nonlinear interactions; consequently, they significantly affect dispersion, residence time, and the analytical signal. Since these parameters are interdependent, joint optimization is essential to maximize sensitivity, maintain linearity, minimize carryover, and reduce reagent consumption. Therefore, a Central Composite Design (CCD) was employed because it efficiently models multivariate systems and captures curvature effects on response surfaces. Moreover, CCD integrates factorial, axial, and center points to estimate linear and quadratic terms, enabling the accurate prediction of optimal conditions. Thus, this approach minimizes experimental runs while providing robust statistical models for sensitivity, precision, and throughput optimization.

The central composite design is divided into three blocks with three center points per block, yielding 33 experimental runs. The blank-corrected absorbances for each assay (*A*_FRAP_ and *A*_ABTS_), recorded with Trolox at 60 μmol L^–1^ as the reference antioxidant, served as the response variables. The independent factors examined were the aspiration volume, carrier flow rate, and concentrations of the FRAP and ABTS^•+^ reagents. Aspiration volume was treated as a single factor because the four segments in the sequence—antioxidant, FRAP, antioxidant, and ABTS^•+^—were varied in unison while maintaining the 38–33–38–33 μL proportion. Carrier flow rate was defined as the velocity at which the stream exited the helical reactor and entered the detector (35 μL s^–1^). FRAP concentration was expressed as a percentage of the stock solution—prepared at a 10:1:1 ratio of 0.005 mol L^–1^ HCl, 10 mmol L^–1^ TPTZ, and 20 mmol L^–1^ FeCl_3_·6H_2_O—designated as 100%. ABTS^•+^ concentration was likewise reported as a percentage of the undiluted radical solution, also defined as 100%. The low, center, and high levels for each factor are listed in Table S4 of the Supporting Information. All runs were randomized within each block to minimize the influence of uncontrolled variables; solutions were prepared on the day of experimentation, and signals were acquired at 593 and 734 nm using dual-wavelength detection. This experimental scheme supplied the data set required to model the hydrodynamic and chemical parameters influencing the analytical response and thereby enabled subsequent multivariable optimization of the combined method.

## Features of the Developed Method

### Calibration Curves

Although the FRAP/ABTS-SIA method determines the antioxidant capacity through two simultaneous measurements, each assay requires its own calibration curve. Accordingly, two independent curves were constructed—one for FRAP and another one for ABTS—using Trolox over the 5–400 μmol L^–1^ range. For every concentration, the blank-corrected absorbances (*A*_FRAP_ and *A*_ABTS_) were recorded and plotted against the corresponding Trolox level. The procedure was repeated on five consecutive days (one curve per day), providing five replicates from which an average calibration curve was obtained for each assay. Limits of detection and quantification were calculated from the residual standard deviation of the regression (*S*_*y*/*x*_) and the mean slope (*b*) following the method described in the literature.^42^

### Intermediate Precision

The precision of the FRAP/ABTS-SIA method was assessed in terms of repeatability (intraday) and intermediate reproducibility (interday) within the same laboratory. On five consecutive days, duplicate aqueous solutions of Trolox (60 μmol L^–1^) and a honey-processed coffee extract (0.5% w/v) were prepared with distilled water and filtered (0.45 μm) before analysis. Each day, both replicates were introduced into the system in three independent aspirations under the optimized hydrodynamic parameters. Antioxidant capacity for every injection was expressed as μmol L^–1^ Trolox equivalents by interpolation on the average calibration curve established for each assay. The resulting data set, organized hierarchically by day and replicate, was subjected to one-way analysis of variance (ANOVA) to partition intraday and interday variability, following the international guidelines and the statistical procedure.^42,43^ Mean squares from the ANOVA provided estimates of the repeatability standard deviation and the intermediate reproducibility standard deviation, yielding a quantitative measure of the method’s overall precision for both the standard and coffee matrices.

### FRAP and ABTS Assays by the Microplate

To compare the antioxidant capacity obtained with the proposed sequential method against the reference procedures for each assay, microplate versions of FRAP and ABTS were carried out using a 96-well absorbance reader (Synergy H1, BioTek) thermostated at 25 °C. Calibration curves were prepared with freshly made aqueous solutions of Trolox; the ABTS curve covered 5–80 μmol L^–1^, whereas the FRAP curve spanned 5–120 μmol L^–1^, ensuring both ranges fell within the linear response region of their respective reactions.

For the FRAP assay, the reagent was prepared according to that published by Bolanos De La Torre et al. in 2015,^44^ with the only modification being to adjust the pH to 3.6. Each well received 280 μL of reagent and 20 μL of sample or standard, yielding a final volume of 300 μL. After a 30 min incubation in the dark, absorbance was measured at 593 nm, and each reading was corrected with the corresponding blank (reagent plus water).

For ABTS, the radical cation was generated ex situ and diluted 1:15 with distilled water to an initial absorbance of approximately 1.0 at 734 nm. Each well was loaded with 150 μL of the radical solution and 150 μL of sample or standard, mixed briefly, and, after 15 min at room temperature, the residual absorbance was recorded at 734 nm,^45^ using the radical solution plus water as the blank.

Net absorbances (*A*_FRAP_ and *A*_ABTS_) were plotted against the Trolox concentration and fitted by weighted linear regression to generate calibration equations. Antioxidant capacities of the samples, expressed as μmol L^–1^ Trolox equivalents, were obtained by direct interpolation, ensuring that absorbance values remained within the linear range of each curve. This approach provided a quantitative basis for comparing the results of the simultaneous method with those derived from the reference microplate protocols.

### Recovery Study

Method accuracy and potential matrix effects were assessed through recovery experiments in which ten representative samples were fortified with Trolox, used as the reference standard. Initially, the intrinsic antioxidant capacity of each sample was measured in triplicate, after dilution with distilled water, to ensure the response fell within the linear range of the calibration curves. The matrices analyzed included Merlot and Carménère red wines (0.25% v/v), commercial cranberry juice (2.50% v/v) and pomegranate juice (0.45% v/v), aqueous extracts of “honey-processed” and “washed” coffee (0.57% v/v), green-tea infusion (0.20% v/v), and macerates of oregano (0.63% v/v), cumin (4% v/v), and rosemary (1.25% v/v). Using the antioxidant capacities obtained, fresh aliquots of each matrix were spiked with 20 μmol L^–1^ Trolox and analyzed under identical conditions. The recovery percentage was calculated as the ratio of the increase in antioxidant capacity—defined as the difference between the fortified and the unfortified sample—to the theoretical Trolox concentration added, multiplied by 100, following the recommendations of international guidelines and statistical procedures.^42,43^ This approach provided a quantitative estimate of the method’s accuracy and the extent of matrix effects for each sample type evaluated.

## Results and Discussion

### Implementation of the ABTS-SIA Assay

The initial step in integrating the FRAP and ABTS assays into a unified SIA protocol involved establishing a reliable ABTS-SIA module with timing, reagent volumes, and detection parameters aligned with those previously optimized for the FRAP-SIA system.^41^

Table S1 and Figure S1 of the Supporting Information depicts the programming and signals of the ABTS-SIA method. The aspiration sequence consisted of ABTS–antioxidant–ABTS, with corresponding aspiration volumes of 33 μL, 38 μL, and 33 μL, respectively.

On the other hand, the concentration of the ABTS^•+^ radical was assessed with the objective of identifying the condition that produced the greatest difference between the absorbance signal of the unreacted ABTS^•+^ radical and that observed after its reaction with an antioxidant. When the ABTS^•+^ stock solution was diluted to a ratio of 1:10 and passed through the detector in the absence of Trolox, a baseline absorbance of 0.492 ± 0.017 A at 734 nm was recorded. Reducing the dilution to 1:5 resulted in an approximate doubling of the optical density to 0.973 ± 0.006 A. Upon introduction of a 400 μmol L^–1^ Trolox solution under identical hydrodynamic conditions, the ABTS^•+^ chromophore was almost completely decolorized, with absorbance values decreasing to 0.028 ± 0.004 A (1:10 dilution) and 0.024 ± 0.006 A (1:5 dilution), indicating near-complete quenching of the radical cation. Since ABTS^•+^ decolorization occurs through a single-electron transfer followed by proton donation, the extent of absorbance reduction is directly correlated with the hydrogen-donating capacity of the antioxidant.^44^ The marked difference between the blank and reacted signals thus confirmed that the online reaction was both rapid and complete.

Selecting the 1:5 dilution as the working concentration delivered two practical advantages: the initial absorbance remained close to unity, maximizing the dynamic window for signal decay, and the peak-to-peak noise decreased, improving the precision in absorbance measurement. At higher dilution (1:10), the radical peak was only half as intense, compressing the useful range and increasing the relative contribution of baseline drift to the overall uncertainty. The choice of a 1:5 dilution is consistent with recent ABTS-based SIA reports that recommend an initial absorbance close to unity to balance sensitivity and detector linearity.^32^

Using the optimized dilution, a calibration curve was constructed by injecting Trolox standards from 10 to 300 μmol L^–1^ in triplicate. The resulting regression, shown in Figure S2 of the Supporting Information, exhibited excellent linearity (*r* ^2^ = 0.993) and homoscedastic residuals. The slope indicates that a 1 μmol L^–1^ change in Trolox produces a 0.0033 A decrease in absorbance under the selected conditions, a sensitivity comparable to or higher than that reported for other high-throughput Total Antioxidant Capacity (TAC) methods employing flow injection published in the last years.^46^ Most importantly, the nearly identical aspiration sequence and holding coil residence time used for both assays pave the way for their seamless fusion into a dual-readout SIA method without introducing additional hardware or programming complexity.

### Optimization of the FRAP/ABTS-SIA Method

The SIA system design was based on optimizing the balance among analytical sensitivity, dispersion control, zone size, resolution between consecutive peaks, and the stability of the aspirated concentration profile.

### FRAP/ABTS-SIA System Signal Evaluation

#### Analytical Signal Behavior without a Coil Reactor

Before the experimental results presented in this section are discussed, two methodological considerations must be emphasized. The simultaneous FRAP/ABTS-SIA protocol was designed to create two discrete reaction zones and, consequently, two independent spectrophotometric peaks; data treatment relied exclusively on the maximum height of each peak, a variable directly proportional to antioxidant capacity. The dual-channel spectrophotometer used in this study allowed simultaneous monitoring at two wavelengths. Although the ABTS^•+^ radical is conventionally quantified at 734 nm and the FRAP reagent at 593 nm, the radical also exhibits appreciable absorbance at 593 nm, whereas the ferric-TPTZ complex is essentially transparent at 734 nm. This feature made it possible to track both reactions at 593 nm during the preliminary trials.

So, the initial experiments aimed to achieve spatial separation between the two reaction zones corresponding to the ABTS and FRAP assays. To this end, a 110 μL water plug was introduced between each reaction segment. Four distinct flow sequences were evaluated, each incorporating the specific reagent volumes: 33 μL for FRAP, 33 μL for ABTS, and 38 μL for the antioxidant solution (Table S2 of the Supporting Information). The sequences of Experiment 1 (FRAP–Antioxidant–FRAP–H_2_O–ABTS–Antioxidant–ABTS–H_2_O) and Experiment 2 (ABTS–antioxidant–ABTS–H_2_O–FRAP–antioxidant–FRAP–H_2_O) produced a single and well-defined peak, corresponding to the ABTS assay, and a second peak very wide for the FRAP assay. This outcome is attributed to the lengthy aspiration sequence, which produced extended reaction zones and, consequently, excessive axial dispersion. This behavior is consistent with Taylor Dispersion models for sequential-injection systems operating with long residence times.^47^ In addition, an analysis of the same aspiration sequence of Experiment 1, with the FRAP reagent replaced by water, confirmed that the broadened signal observed at the end of the FIAgram was exclusively associated with the FRAP assay, as this signal disappeared in the absence of the reagent (Figures S3 and S4 of the Supporting Information). The other two sequences, Experiment 3 (FRAP–antioxidant–H_2_O–antioxidant–ABTS), and Experiment 4 (ABTS–antioxidant–H_2_O–antioxidant–FRAP) exhibited the same dispersion phenomenon and the order in which peaks appeared (Figures S5 and S6 of the Supporting Information).

These results demonstrate that the reaction kinetics of the ABTS assay is faster than those of the FRAP assay, as the first peak consistently corresponds to ABTS regardless of the aspiration order. Shah and Modi^48^ previously showed that reaction times for ABTS range from 1 to 6 min, whereas the FRAP batch assay requires approximately 60 min to reach completion. Their findings confirmed that ABTS exhibits faster reaction kinetics than FRAP. More recently, Villar Idoate et al.^49^ demonstrated not only that the ABTS method reacts more quickly than FRAP but also that the presence of additional hydroxyl groups significantly enhances the antioxidant capacity in ABTS, whereas substitution with methoxy groups reduces the antioxidant activity in FRAP and DPPH, but not in ABTS.

Considering these findings, a reactor coil was placed before the detector, allowing the FRAP reaction bolus to remain in place while the second part of the sequence—aspiration of the ABTS reagents—was carried out.

### Impact of the Reaction Coil Integration on the Analytical Signal

The preliminary results demonstrated the need to optimize the signals (peaks) generated using the simultaneous method, as it was not possible to adequately control the dispersion of the aspirated boluses characteristic of each assay having insufficient reaction time for the FRAP method, resulting in one defined peak and another peak broadened significantly. Furthermore, recent studies suggest that minimizing liquid–liquid interfaces in flow systems is an effective strategy to reduce dispersion.

### Dispersion Control

Based on these findings, the Experiments in this section aimed to shorten the aspiration sequence while extending the reaction time for the FRAP assay. To accomplish this, a revised programming and configuration scheme was implemented for the sequential injection analysis (SIA) system. This included the integration of a 635 μL helical reaction coil, positioned directly before the detector. This configuration eliminates the need for aspirating a water bolus between assays and enables the FRAP reagents to be aspirated first and directed into the helical reactor, initiating the reaction while the ABTS reagents are subsequently aspirated. This modification enabled two significant advances. First, it was possible to aspirate the first bolus composed of the antioxidant and the FRAP reagent, which was directed into the reactor coil and retained for a few seconds, starting the reaction, allowing more time for the FRAP reaction. Simultaneously, a second bolus containing the antioxidant and the ABTS reagent was aspirated, thus preventing mixing between the two and allowing them to pass through the detector as clearly separated signals. Second, the temporary retention of the first pellet in the reactor also allowed its dispersion to be controlled. Temporarily halting the flow yielded sharp, well-defined peaks with minimal broadening and high reproducibility, as illustrated in Figure 2a.

**Figure 2:**
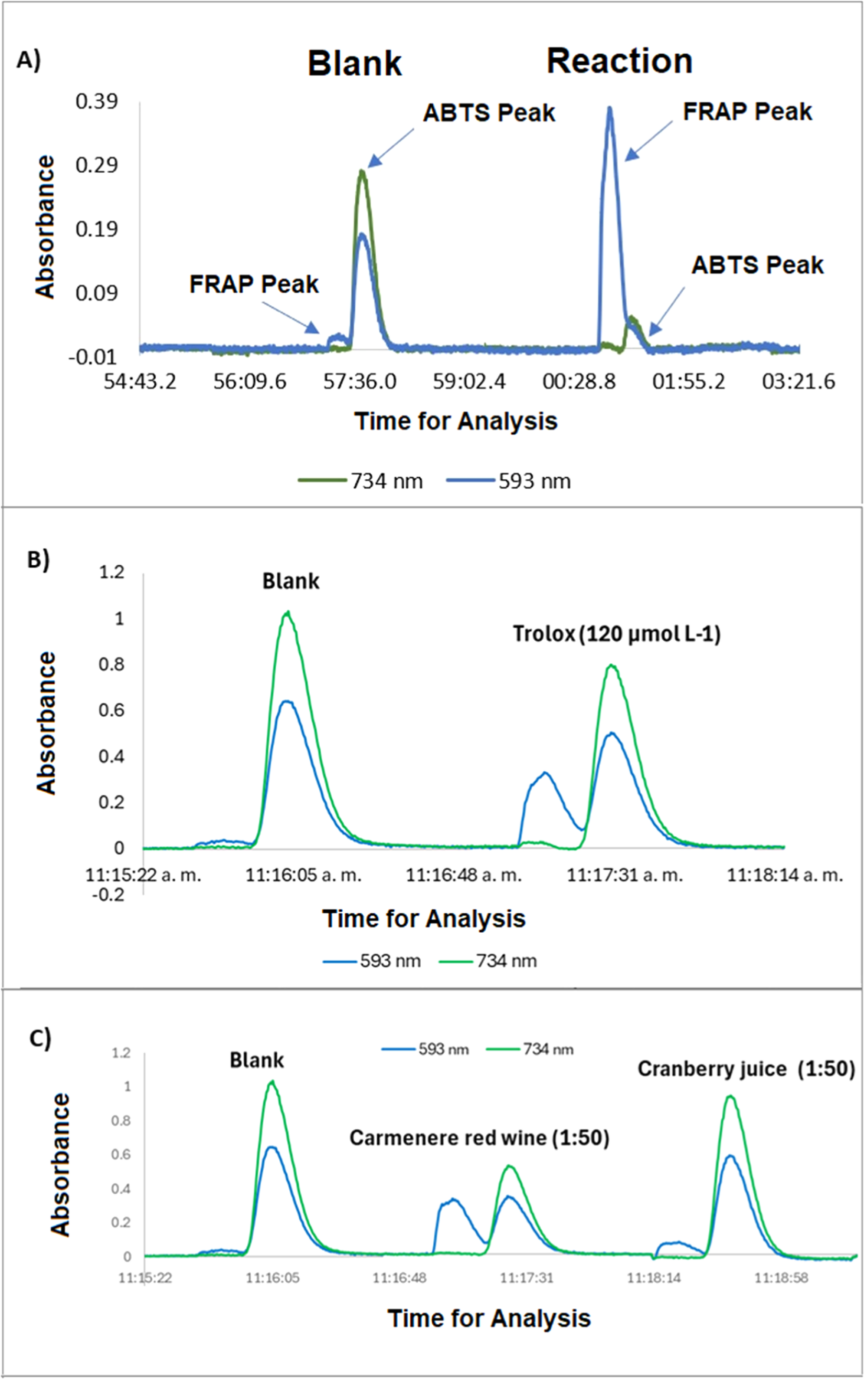
Diagram of the reaction signal of the SIA system, using the ABTS and FRAP assays simultaneously against Trolox 140 μmol L^–1^ before optimal conditions (A), Trolox 120 μmol L^–1^ (B) and samples (C) after optimal conditions. The samples Carmenere red wine and cranberry juice were diluted 50-fold with distilled water.

Thus, a feasible preliminary aspiration sequence was established for the simultaneous SIA system, in the order antioxidant–FRAP–(dispensed reactor coil)–antioxidant–ABTS. Injection of blank (distilled water) produced two peaks at 593 nm: the first, 0.027 ± 0.006 A, corresponded to the FRAP reagent and the second, 0.184 ± 0.004 A, to the ABTS^•+^ radical, which, although maximally absorbed at 734 nm, also exhibits visible absorption. At 734 nm, only one peak was detected (0.285 ± 0.006 AU), assigned to the ABTS^•+^ blank. The reactor’s dual role—as a temporary reservoir and as a radial/axial diffusion mixer—accounted for this separation: while the FRAP product developed, the ABTS segment remained in the holding coil, and both boluses traversed under laminar flow, reducing axial dispersion as predicted by dispersion models.

Because the ABTS^•+^ peak intensity decreased by 35% when monitored at 593 nm, the detector’s multichannel capability was exploited: the FRAP peak was measured at 593 nm and the ABTS peak at 734 nm. When the same sequence was run with 140 μmol L^–1^ Trolox concentrations, the first peak grew proportionally whereas the second diminished, confirming the expected kinetics for both assays.^50^ Similar findings—where peak separation is critically dependent on reactor design and intersegment spacing—have been reported for high-throughput antioxidant determinations in sequential-injection and FIA systems.^51^

### Optimized Aspiration Sequence

The preceding results suggest that the FRAP reaction requires a longer reaction time. Consequently, four aspiration sequences were designed to evaluate the order of reagent and antioxidant introduction—both in the FRAP and ABTS assays—using Trolox at a concentration of 60 μmol L^–1^. In these sequences, the FRAP and antioxidant reagents were aspirated first and directed into the reaction coil, followed by the aspiration of ABTS and the antioxidant toward the holding coil. Table S5 in the Supporting Information summarizes the tested sequences and the corresponding absorbance peak heights.

To enhance the bullet-shaped parabolic profile of the final aspirated bolus—thereby stabilizing the concentration gradient and minimizing axial dispersion—further experiments were conducted using Trolox at 120 μmol L^–1^. An additional step was introduced in the aspiration sequence: 100 μL of distilled water was aspirated into the holding coil following the ABTS-antioxidant bolus prior to delivery to the detector. This modification significantly mitigated the potential tailing effect, aligning with recommended strategies for optimizing the separation zone between reactions in sequential injection analysis systems.

The selection of the antioxidant–FRAP (dispensed reactor coil)–ABTS–antioxidant–water sequence yielded FRAP and ABTS values of 0.271 ± 0.002 and 0.197 ± 0.003, respectively.

Therefore, the SIA system collector operated in two hydrodynamic stages. In the first, the segments corresponding to the antioxidant and FRAP reagent were aspirated and propelled toward the reactor to allow their mixing and reaction. In the second stage, the segments containing the ABTS^•+^ radical, the antioxidant, and 100 μL of water were loaded and sent to the same reactor, which already contained the FRAP reaction product. This strategy allowed both reaction pellets to be transported consecutively to the detector, avoiding their intermixing and guaranteeing independent signals.

This configuration also addresses axial dispersion, a phenomenon that broadens zones and merges signals due to velocity gradients in the tubing. The helical reactor promotes radial diffusion and reduces the axial gradients, as predicted by laminar dispersion models. Consequently, the aspiration order reflects intrinsic kinetic, spectral, and hydrodynamic properties, ensuring the accurate and independent detection of both antioxidant assays within sequential injection analysis systems.

### Central Composite Design (CCD) for FRAP/ABTS-SIA System Optimization

To identify the factors that exert a significant effect on the FRAP/ABTS-SIA method and, consequently, to establish its optimal conditions, a central composite design (CCD) was applied with two independent response variables: the absorbance of the FRAP assay (A_FRAP_) and that of the ABTS assay (*A*_ABTS_). The experimental matrix and individual results are detailed in Table S6 of the Supporting Information.

Analysis of variance (ANOVA) indicated that, for both response variables, the total aspiration volume and the concentration of the ABTS^•+^ radical had statistically significant effects (*p* < 0.05), while the remaining factors showed minimal or nonsignificant influence. Nevertheless, the Pareto charts (Figures S7 and S8 in the Supporting Information) revealed contrasting trends regarding the concentrations of the specific reagents: in the FRAP model, higher FRAP reagent concentrations and lower ABTS^•+^ concentrations were favored; in contrast, the ABTS model exhibited the opposite preference. This antagonistic behavior aligns with the reaction kinetics and extinction coefficients of the two assays, as reported in other flow-based antioxidant capacity methodologies.^26,32,40,52,53^

Each analysis of the design based on its response variable proposed optimal values for each evaluated factor, showing differences in the optimal values for the FRAP reagent and the ABTS radical. However, the optimal values suggested for the flow rate (40 μL s^–1^) and aspiration volume (antioxidant (43 μL)–FRAP (38 μL)–ABTS (38 μL)–antioxidant (43 μL)–water (100 μL)) were the same in each design analysis, so these values were considered as optimum. In flow systems, large volumes increase dispersion and compromise peak separation, whereas volumes that are too small reduce sensitivity by generating poorly defined zones. The use of symmetric volumes around the antioxidant improves the parabolic zone geometry and reduces distortion, as recommended for antioxidant determinations in FIA systems.^15,36,50^

The optimal flow rate of 40 μL s^–1^ was selected because: (1) lower flow rates not only increase residence time but also enhance axial dispersion; and (2) higher flow rates reduce the effective reaction time for FRAP. This compromise was identified through the results of the central composite design (CCD), where flow rate proved significant for both FRAP and ABTS responses.

To achieve a balanced reagent concentration, three additional experiments were conducted (Table S7 of the Supporting Information), in which the flow rate and aspiration volumes were fixed at their found optimal values, while only the relative concentrations of FRAP and ABTS^•+^ were varied, using 60 μmol L^–1^ Trolox as the standard. The optimal results were obtained using a 70% concentration for the FRAP reagent and a 50% concentration for the ABTS^•+^ radical, which were adopted as the final operating conditions. This combination represents a balanced compromise that maximizes the sensitivity of both assays while aligning with response surface methodology recommendations for multiresponse systems. Optimized signals are shown in Figure 2b.

Based on the results obtained from the central composite design, clearly differentiated trends were observed for each of the evaluated responses. For the FRAP assay, the model indicated that system sensitivity increased with higher concentrations of ferric reagent and decreased with concentrations of the ABTS^•+^ radical, which is consistent with the slow kinetics of the Fe­(III)/Fe­(II) reduction mechanism. In contrast, the ABTS response exhibited an opposite dependence: higher ABTS^•+^ concentrations provided a broader dynamic range and a more stable signal–concentration relationship.

These differences were reflected in the generated response surfaces, whose interpretation required the adoption of a multiresponse optimization approach using the joint desirability criterion recommended for dual-signal analytical methods.^42^ The combination of factors that provided the best compromise between both response curves corresponded to 70% of the FRAP reagent concentration and 50% of the ABTS^•+^ radical concentration while maintaining constant the previously optimized flow rate (40 μL s^–1^) and aspiration scheme, both shown to simultaneously reduce axial dispersion and enhance separation between analytical peaks. This balanced selection agrees with prior studies showing that combined Electron Transfer-based methods require differential adjustment of oxidants and reductants to maintain an appropriate balance between sensitivity and signal definition.^26^

Finally, the role of total aspirated volume in shaping the parabolic profile of the zone and managing axial dispersion was highlighted, as this factor was statistically significant in both models (*A*_FRAP_ and *A*_ABTS_), an effect theoretically expected according to Taylor’s hydrodynamic behavior and experimentally validated in high-flow coiled-tubing systems.^47^

Figure 3 shows the performance attributed to the central location of both reagents in the aspiration sequence, separated by an approximate volume of 519 μL of carrier water. This volume, plus 38 μL of FRAP reagent and 43 μL of antioxidant, forms the first 600 μL bolus that is sent to the helical reactor. This configuration favored efficient radial mixing among the reagents, the sample, and the carrier, which prolonged the reaction time and, consequently, increased the magnitude of the generated peak. In addition, the selected sequence offers the best compromise between the controlled dispersion and analytical sensitivity.

**Figure 3:**
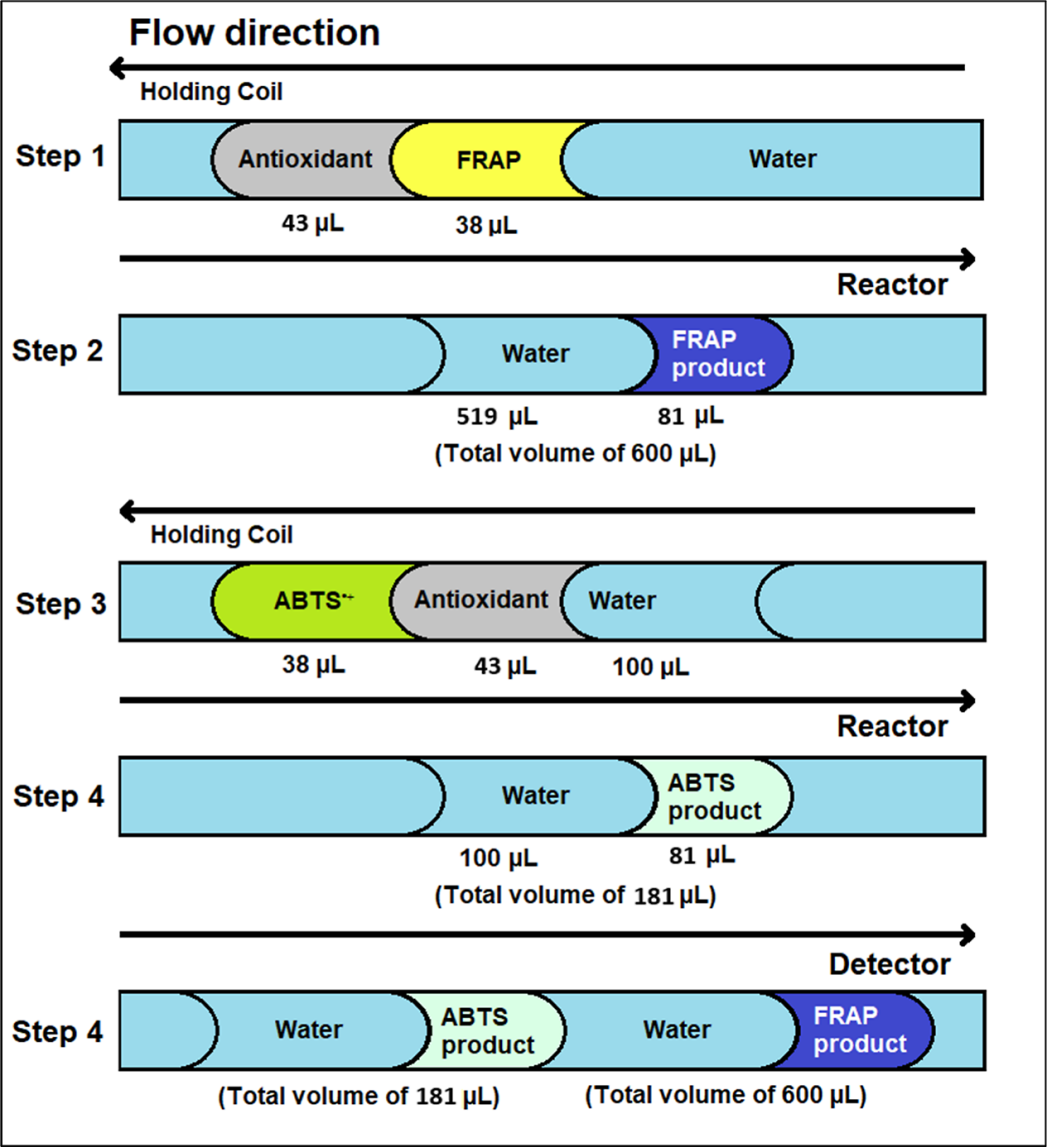
Optimal conditions for sequential aspiration programming: antioxidant–FRAP–antioxidant–ABTS, with volumes of 43–38–43–38 μL and carrier flow of 35 μL s^–1^.

### Validation Parameters

Method validation included evaluation of linearity through five independent calibration curves, determination of LOD and LOQ based on *S*_*y*/*x*_ and slope values, assessment of repeatability and intermediate precision, recovery studies in real matrices, and statistical comparison with a reference microplate method. Collectively, these results confirm the analytical reliability and applicability of the proposed system for antioxidant capacity screening.

### Calibration Curves FRAP/ABTS-SIA

To evaluate the linearity of the simultaneous method, the absorbances of both assays were plotted against Trolox standard solutions ranging from 5 to 400 μmol L^–1^. The FRAP assay response was linear up to 150 μmol L^–1^, whereas the ABTS assay maintained linearity throughout the entire interval. To establish a common range—indispensable when both determinations are performed in the same run—an operational interval of 10–120 μmol L^–1^ was selected for both assays. This ensures that any sample analyzed can be diluted in a single linear range.

To obtain robust estimates of slopes and intercepts, an independent calibration curve for each assay was constructed daily over five consecutive days; the data were combined to generate an average curve used for routine determinations. The resulting regression lines are shown in Figures S9 of the Supporting Information for FRAP assay and Figure S10 of the Supporting Information for ABTS assay.

For the FRAP assay, linear regression yielded a slope of 0.0026 ± 0.0001, an intercept of 0.0060 ± 0.0065, and a correlation coefficient (*r*) of 0.9979. The limit of detection (LOD) and limit of quantification (LOQ) were 0.031 μmol L^–1^ and 0.091 μmol L^–1^, respectively, at the 95% confidence level.

The ABTS assay curve showed a slope of 0.00230 ± 0.00002, an intercept of −0.0094 ± 0.0014, and a *r* = 0.9999. The calculated LOD and LOQ were 0.0047 μmol L^–1^ and 0.0062 μmol L^–1^, respectively. Although the FRAP slope is slightly higher, indicating a greater response per concentration unit, the lower residual variance of the ABTS assay confers lower detection and quantification limits.

Overall, the two average curves exhibit high linearity and suitable sensitivity parameters for quantifying antioxidant capacity within the selected range, thereby laying the groundwork for the analytical application of the combined FRAP/ABTS-SIA method.

### FRAP/ABTS-SIA System Intermediate Precision

The precision of the FRAP/ABTS-SIA system was assessed through intraday (repeatability) and interday (intermediate precision) studies, using a Trolox standard solution (60 μmol L^–1^) and an aqueous “honey” coffee extract at 0.5% w/v as analytical models, thereby allowing performance to be compared in a simple matrix and in a complex one. Tables S8 and S9 of the Supporting Information list the antioxidant capacities obtained—expressed as μmol L^–1^ Trolox equivalents—and the derived statistics. For the FRAP assay, coefficients of variation were 1.14% (repeatability) and 1.89% (intermediate precision) for the standard solution and were 1.70% and 2.04% for the coffee extract. On the other hand, the ABTS assay showed 0.87% and 2.09% with Trolox and 0.78% and 1.82% with the coffee extract. All values fall below the 2% threshold recommended by the AOAC and Codex Alimentarius for laboratory methods.^54,55^ The absence of significant differences between the precision achieved with the pure compound and with the complex matrix confirms that the simultaneous procedure remains robust against potential sample interferences, thereby establishing it as a reliable tool for the routine quantification of antioxidant capacity.

### Calibration Curves of FRAP and ABTS Assays in Microplate

The application of the FRAP and ABTS assays in microplate format enabled the generation of their respective calibration curves with Trolox standard solutions and, subsequently, the quantification of antioxidant capacity in various food matrices. For the FRAP assay, a linear calibration line was obtained between 5 and 120 μmol L^–1^ (slope 0.00210 ± 0.00004 and intercept −0.0032 ± 0.0027), whereas the ABTS assay exhibited linearity from 5 to 80 μmol L^–1^ (slope 0.0096 ± 0.0001 and intercept 0.0087 ± 0.0061). These ranges agree with those reported for microplate methods based on FRAP and ABTS^•+^, respectively.^56,57^

### Analysis of Samples

Ten food matrices were evaluated using the simultaneous FRAP/ABTS-SIA method (Figure 2c) and, independently, the microplate FRAP and ABTS assays to determine antioxidant capacity as Trolox equivalents (μmol L^–1^). A paired two-tailed *t* test (α = 0.05) was applied to compare the SIA method with the reference microplate assay, as no a priori directional bias was assumed between methodologies. Differences may occur in either direction due to variations in hydrodynamics or effective reaction time; therefore, a two-tailed approach is statistically appropriate.^40^ Comparative results are presented in Table 2. The test showed significant differences between the values obtained with the SIA platform and those determined by microplate, implying that absolute magnitudes are not interchangeable without prior cross-calibration.

**Table 2:** Results of Antioxidant Capacity in Different Food Samples by the FRAP/ABTS-SIA Method and the Microplate Method

	ABTS		FRAP	
samples	SIA^a^	LOTE^a^	SIA^a^	LOTE^a^
honey coffee extract	6830.07 ± 430.41	8507.20 ± 321.38	6591.95 ± 536.46	8523.48 ± 1137.97
washed coffee extract	7616.30 ± 343.10	8022.79 ± 63.79	5906.13 ± 299.28	7145.51 ± 170.96
green tea extract	24949.28 ± 307.44	25 897.93 ± 403.14	13 429.75 ± 670.43	18919.64 ± 406.42
cranberry juice	1822.03 ± 155.88	1652.47 ± 105.15	1203.33 ± 26.28	1866.33 ± 47.24
pomegranate juice	9447.25 ± 438.33	10984.03 ± 212.19	6924.23 ± 315.88	10819.17 ± 412.98
Carmenere red wine	16655.07 ± 491.90	17341.30 ± 291.63	11502.31 ± 613.10	15765.65 ± 573.48
Merlot red wine	16742.03 ± 482.70	17133.69 ± 157.20	9833.59 ± 546.97	14946.73 ± 525.30
oregano extract	6546.09 ± 263.37	8752.37 ± 489.97	5763.97 ± 70.07	7036.98 ± 198.66
cumin extract	1022.83 ± 34.94	1477.42 ± 31.57	894.30 ± 68.37	1280.63 ± 6.77
rosemary extract	3244.06 ± 243.48	3340.93 ± 95.77	3028.63 ± 139.04	3846.05 ± 108.38

a Antioxidant capacity values expressed in μmol L^–1^ of Trolox.

Nonetheless, when the antioxidant capacities derived from both procedures were plotted (Figure S11 of the Supporting Information), a high linear correlation was observed: *r* = 0.98 for FRAP and *r* = 0.99 for ABTS. This level of association indicates that, although systematic offsets exist—likely attributable to differences in hydrodynamics, residence times, and reagent stoichiometries—the trends among samples are preserved, allowing their relative comparison. Similar findings, in which agreement is expressed through high correlation despite biases in absolute values, have been reported when flow methods are contrasted with microplate protocols for antioxidant-capacity quantification.^41,46,58^

Practically, the excellent intermethod linearity demonstrates that the FRAP/ABTS-SIA system is well suited for rapid screening and comparative ranking of foods according to their reducing power and radical-scavenging capacity while offering higher analytical throughput, lower reagent consumption, and shorter analysis times than conventional microplate methodology.

To evaluate the agreement between the microplate methodology and the simultaneous SIA system, absorbances obtained with both procedures at identical Trolox levels were plotted; Figure S12 of the Supporting Information shows a correlation coefficient of 0.99 for each assay, confirming the analytical equivalence of the two platforms and supporting the reliability of the antioxidant-capacity values determined by the FRAP/ABTS-SIA system.

The slope of the FRAP correlation line was approximately five times greater than that of the ABTS assay. This difference is attributed, on the one hand, to the use of a lower-than-usual FRAP reagent concentration, which enhances sensitivity by avoiding saturation of the Fe­(II)-TPTZ complex, and, on the other hand, to the need to increase the ABTS^•+^ radical concentration to ensure an initial absorbance close to 1, thereby reducing the slope because a smaller fraction of radical can be decolorized. Even so, the high correlation obtained indicates that adjusting reagent concentrations does not compromise the relative precision between methods but rather optimizes the linear window for simultaneous operation.

The selected samples encompass four food categories: wines, natural products, processed juices, and culinary condiments, each subjected to different treatments. Consequently, the results demonstrate that the FRAP-ABTS-SIA method can be applied to food matrices of diverse origins and may be extrapolated to other types of food products.

### Evaluation of Recoveries of the FRAP/ABTS-SIA Assay

The primary aim of this work was the development of a sequential injection system capable of performing FRAP and ABTS assays simultaneously within a single analytical cycle. Accordingly, matrix effects were evaluated through recovery studies in representative food matrices rather than through isolated interferent testing. This approach is consistent with international validation guidelines^43,54^ (EURACHEM 2016; AOAC Appendix K) as recovery assays integrate the combined effect of matrix constituents commonly present in real samples.

The recovery percentages obtained with the FRAP/ABTS-SIA configuration are summarized in Table 3, ranging from 97.34 to 106.19% for FRAP and from 92.75 to 105.43% for ABTS. The mean values (100.7 ± 3.2% and 99.1 ± 4.1%, respectively) do not differ significantly from 100% at the 95% confidence level (two-tailed *t*-test), demonstrating the absence of systematic bias and confirming the method’s accuracy. These recoveries lie within the 80–110% acceptance window set by the AOAC and Codex Alimentarius for routine methods applied to complex food matrices.^54,55^

**Table 3:** Recovery Percentages Obtained for the FRAP Test by the FRAP/ABTS-SIA System^a^

samples	% *R* FRAP	% *R* ABTS
honey coffee extract (0.57%)	101.13	101.09
washed coffee extract (0.57%)	102.08	97.83
green tea extract (0.20%)	97.66	103.26
cranberry juice (2.50%)	102.40	105.43
pomegranate juice (0.45%)	104.30	98.91
Carmenere red wine (0.25%)	106.19	102.90
Merlot red wine (0.25%)	104.30	103.26
oregano extract (0.63%)	100.19	99.28
cumin extract (4.00%)	97.34	92.75
rosemary extract (1.25%)	98.92	96.01

a %*R*: recovery percentage.

The slightly wider dispersion observed in the ABTS assay—somewhat greater than that in FRAP—can be attributed to the higher susceptibility of the ABTS^•+^ radical to high-molecular-weight phenolics present in the extracts, a phenomenon previously described for antioxidant capacity methods.^26^ Nevertheless, even the lowest value of 92.75% exceeds the 90% threshold regarded as indicative of negligible matrix interference, confirming that the matrix does not compromise the determination.

Compared with other high-throughput methods—such as microplate or conventional FIA—the procedure described here affords equal or superior recoveries, along with shorter analysis times and lower reagent consumption, advantages inherent to sequential injection analysis. Taken together, the results substantiate the robustness of the system for the routine quantification of antioxidant capacity in both pure standards and compositionally complex samples.

On the other hand, despite the analytical advantages of the proposed FRAP/ABTS-SIA system (speed, reagent economy, metrological robustness, and simplified data processing), certain limitations should be acknowledged. The implementation requires dedicated flow instrumentation and programming expertise (a high-precision bidirectional pump, a multiselection valve, compatible spectrophotometric detectors, and electronic flow controllers), which may represent a higher initial cost compared to conventional batch assays. Furthermore, hydrodynamic optimization demands careful control of the aspiration order, flow rate, and residence time, making the approach particularly suitable for laboratories experienced in flow-based analysis. The hydrodynamic optimization involves considering aspiration order, zone geometry, flow rates, sequential volumes, residence time in the reactor, and control of the axial dispersion. These parameters interact with one another and must be adjusted carefully to avoid signal overlap or nonlinear effects.

Also, it is relevant to mention that the ABTS-SIA calibration curve has a slope lower than ABTS-microplate, so also it is lower sensitive, but this did not affect the reproducibility, recoveries, and precision of method.

## Conclusions

Considering the reaction kinetics of each assay and effectively controlling dispersion, a novel procedure was developed using a sequential injection analysis (SIA) system capable of simultaneously quantifying antioxidant capacity via both the ABTS and FRAP assays. This dual-assay approach yields two independent analytical signals within a single 2 min run, decreasing considerably the analysis time for two antioxidant capacity assays. Also, the optimized hydrodynamic parameters reduce both sample and reagent volumes, as well as their concentrations, thereby lowering chemical consumption and waste generation. A further methodological advantage is that quantification is based on peak height rather than integrated area; this strategy streamlines data handling, minimizes the influence of axial dispersion, and allows accurate resolution of partially overlapped signals without compromising antioxidant-capacity results, as confirmed by the high correlation coefficients obtained against microplate methods. Once optimized, the system behaves with high reproducibility, and the programming and configuration can be reused for multiple sample types without requiring additional adjustments. The system exhibits intralaboratory precision ≤2% RSD, surpassing the reproducibility typically reported for batch assays, which are often limited by pipetting errors and lengthy incubation times. Altogether, the combination of speed, reagent economy, metrological robustness, and simplified data processing positions the FRAP/ABTS-SIA method as an efficient and reliable analytical tool for the routine evaluation of antioxidant capacity in food and biomedical matrices.

The dual SIA system offers significant advantages, including reduced analytical time, lower dispersion, automation, reduced reagent consumption, and the unique capability to obtain two antioxidant signals simultaneously.

## Supplementary Material



## Notes

SIA system programming, evaluation of aspiration sequences, experiments for choosing the aspiration sequence, central composite design, FIAgram of the reaction signals of the ABTS method by SIA, calibration curve of the ABTS method by SIA, FIAgram of experiment 1, FIAgram of the blank from experiment 1, FIAgram of experiment 3, FIAgram of experiment 4, results for the choice of the aspiration sequence, DCC in 3 blocks with 3 central points, standardized Pareto chart, experiments to determine optimal concentration values, average curve for antioxidant capacity determination for the FRAP assay, results for measuring the precision of antioxidant capacity quantification, correlation of antioxidant capacity, and correlation of absorbances (PDF)
