# Supplementary material for: A Breakthrough SIA-Based Dual Assay for Simultaneous Evaluation of Antioxidant Capacity via ABTS and FRAP Mechanisms
Source: Anal Chem. 2026 Feb 4;98(6):4548–61. doi: 10.1021/acs.analchem.5c05489 (PMC12921658; doi:10.1021/acs.analchem.5c05489)
Supplement: Supplementary file 1 [file ac5c05489_si_001.pdf]

Supporting Information

A Breakthrough SIA-Based Dual Assay for Simultaneous Evaluation of Antioxidant Capacity via ABTS and FRAP Mechanisms

Willmann Antonio Jiménez Morales[1*], María del Pilar Cañizares Macias[2*].

[1] Universidad Autónoma “Benito Juárez” de Oaxaca (UABJO), Facultad de Ciencias Química, Av. Universidad S/N, Cinco Señores, C.P. 68120, Oaxaca de Juárez, Oaxaca, México.
[2] Departamento de Química Analítica, Facultad de Química, Universidad Nacional Autónoma de México, Av. Universidad 3000, Ciudad de México, C.P. 04510, México.

*Corresponding author1: email: wjimenez.fcq@uabjo.mx
*Corresponding author2: email: pilarm@unam.mx

The Supporting Information provides a detailed description of the experimental optimization and validation of the FRAP/ABTS-SIA system. It includes the SIA programming steps for the individual ABTS-SIA method, evaluation of aspiration sequences for simultaneous FRAP and ABTS determinations, and the full central composite design used to optimize hydrodynamic and chemical parameters. Additional figures illustrate representative FIAgrams, calibration curves, Pareto charts, and response correlations between the proposed SIA method and reference microplate assays. Supplementary tables report aspiration sequences, optimization matrices, precision studies, recovery experiments, and statistical parameters supporting method performance and robustness. This material complements the main text by providing comprehensive methodological details and extended datasets that ensure reproducibility and transparency of the proposed analytical approach.

INDEX

Supporting Information	Page
**Table S1.** SIA system programming to determine antioxidant capacity only by the ABTS assay.	S3
**Table S2.** Evaluation of aspiration sequences to develop the simultaneous FRAP/ABTS-SIA method.	S3
**Table S3.** Experiments for choosing the aspiration sequence. Aspiration volumes: ABTS and FRAP 38 µL and for antioxidant 33 µL, and a flow rate of 35 µL s^{-1}.	S4
**Table S4.** Central composite design in 3 blocks with 3 center points per block, to optimize the FRAP/ABTS-SIA system**.**	S4
**Figure S1.** FIAgram of the reaction signals of the ABTS method by SIA, using a trolox standard at 400 µmol L^{-1}.	S4
**Figure S2.** Calibration curve of the ABTS method by SIA, using a trolox standard in a range of 10 to 300 µmol L^{-1}.	S5
**Figure S3.** FIAgram of experiment 1 (sequence FRAP–Antioxidant–FRAP–H_2O–ABTS–Antioxidant–ABTS–H_2O) using trolox at 10 µmol L^{-1}.	S5
**Figure S4.** FIAgram of the blank from experiment 1 (sequence H_2O–Antioxidant–H_2O–H_2O–ABTS–Antioxidant–ABTS–H_2O) using trolox at 10 µmol L^{-1}.	S6
**Figure S5.** FIAgram of experiment 3 (sequence FRAP–Antioxidant–H_2O–Antioxidant–ABTS) using trolox at 10 µmol L^{-1}.	S6
**Figure S6.** FIAgram of experiment 4 (sequence ABTS–Antioxidant–H_2O–Antioxidant–FRAP) using trolox at 10 µmol L^{-1}.	S7
**Table S5.** Results for the choice of the aspiration sequence, based on the estimated absorbance for each assay using trolox at 60 µmol L^{-1}. For all sequences after aspirating FRAP and antioxidant this bolus was sending to reactor.	S7
**Table S6.** DCC in 3 blocks with 3 central points, to evaluate the factors FRAP concentration, $ABTS^{\bullet+}$ radical concentration, aspirate volume and flow rate of the FRAP/ABTS-SIA system, using AFRAP and ABTS as response variables and trolox 60 µmol L^{-1} as standard.	S8
**Figure S7.** Standardized Pareto chart with binary interactions of the factors, to maximize the response of the FRAP/ABTS-SIA system, using AFRAP as the response variable.	S9
**Figure S8.** Standardized Pareto chart with binary interactions of the factors, to maximize the response of the FRAP/ABTS-SIA system, using AABTS as the response variable.	S9
**Table S7.** Experiments to determine optimal concentration values for the FRAP reagent and the $ABTS^{\bullet+}$ radical using trolox at 60 µmol L^{-1}.	S9
**Figure S9.** Average curve for antioxidant capacity determination for the FRAP assay using the FRAP/ABTS-SIA system.	S10
**Figure S10.** Average curve for antioxidant capacity determination for the ABTS assay using the FRAP/ABTS-SIA system.	S10
**Table S8.** Results for measuring the precision of antioxidant capacity quantification in Trolox and honey coffee extract for the FRAP assay by FRAP/ABTS-SIA.	S10
**Table S9.** Results for measuring the precision of antioxidant capacity quantification in Trolox and honey coffee extract for the ABTS assay by FRAP/ABTS-SIA.	S11
**Figure S11.** Correlation of antioxidant capacity (AC) of FRAP and ABTS assays, by FRAP/ABTS-SIA and microplate for 10 food samples.	S11
**Figure S12.** Correlation of absorbances of the FRAP and ABTS assays, by the FRAP/ABTS-SIA system vs microplate when using Trolox concentrations of 10 to 120 µmol L^{-1} for the FRAP assay and 10 to 100 µmol L^{-1} for the ABTS assay.	S12

Table S1. SIA system programming to determine antioxidant capacity only by the ABTS assay.

step	Action	Port	Description	Flow rate (µL s^{-1})	Flow direction	Time (s)
1	Pump filling with carrier	6-7	The pump is filled with 1.5 mL of distilled water.	100	Reverse	15
2	Aspirated ABTS solution	3	33 µL of ABTS solution is aspirated into the holding coil.	70	Reverse	0.47
3	Antioxidant aspirate	4	38 µL of the antioxidant is aspirated into the holding coil.	70	Reverse	0.54
4	Aspirated ABTS solution	3	33 µL of ABTS solution is aspirated into the waiting loop.	70	Reverse	0.47
5	Carrier aspirate	5	110 µL of water is aspirated into the holding coil with 1 s hold.	50	Reverse	3.2
6	Dispensed towards the detector *	2	100 µL is dispensed to the detector and 20 s is waited.	20	Forward	25
7	Dispensed towards the detector r	2	1614 µL is dispensed from the holding coil and pump to the detector	35	Forward	46

* Spectrophotometer set at 734 nm.

Table S2. Evaluation of aspiration sequences to develop the simultaneous FRAP/ABTS-SIA method.

Experiments	Aspiration sequence	Aspiration volume (µL)
1	FRAP–Antioxidant–FRAP–H_2O–ABTS–Antioxidant–ABTS–H_2O	33-38-33-110-33-38-33-110
2	ABTS–Antioxidant–ABTS–H_2O–FRAP–Antioxidant–FRAP–H_2O	33-38-33-110-33-38-33-110
3	FRAP–Antioxidant–H_2O–Antioxidant–ABTS	33-38-110-38-33
4	ABTS–Antioxidant–H_2O–Antioxidant–FRAP	33-38-110-38-33

Table S3. Experiments for choosing the aspiration sequence. Aspiration volumes: ABTS and FRAP 38 µL and for antioxidant 33 µL, and a flow rate of 35 µL s^{-1}.

Experiments	Aspiration sequence
1	FRAP–Antioxidant-ABTS-Antioxidant
2	Antioxidant-FRAP-Antioxidant-ABTS
3	FRAP–Antioxidant-Antioxidant-ABTS
4	Antioxidant-FRAP-ABTS-Antioxidant

Table S4. Central composite design in 3 blocks with 3 center points per block, to optimize the FRAP/ABTS-SIA system**.**

Factors	Low level	High level
Concentration of FRAP reagent (%)	80	100
Concentration of the $ABTS^{\bullet+}$ radical (%)	20	50
Aspirate volume in µL (Antioxidant-FRAP-ABTS-Antioxidant)	28-23-23-28	38-33-33-38
Flow rate (µL s^{-1})	25	35
Total experiments	33	

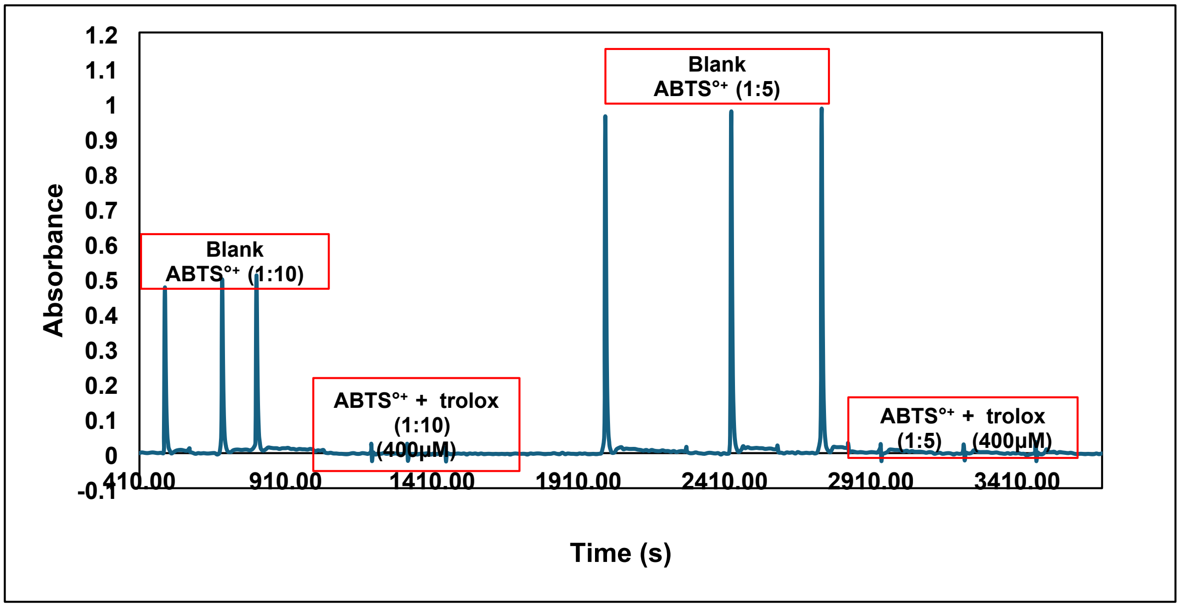

Figure S1. FIAgram of the reaction signals of the ABTS method by SIA, using a trolox standard at 400 µmol L^{-1}.

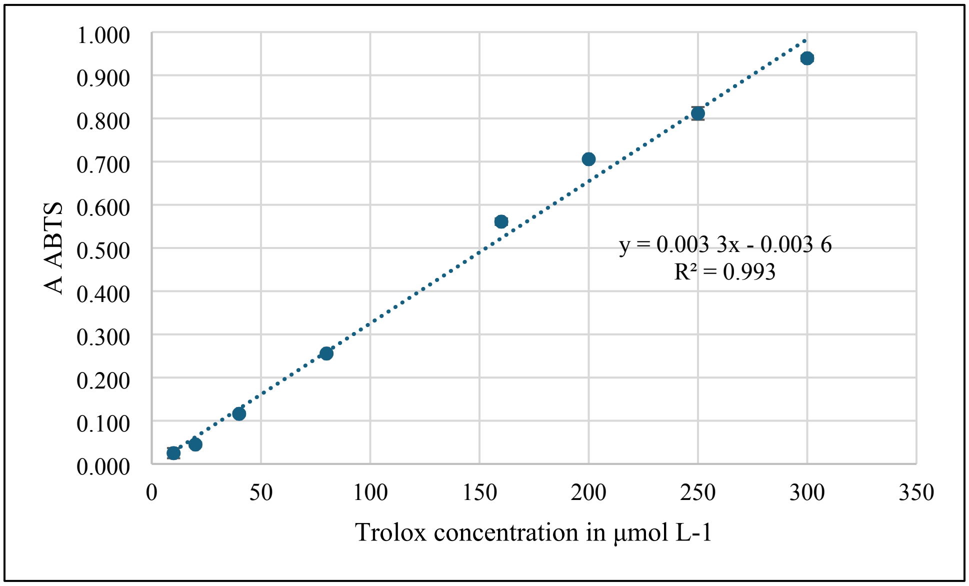

Figure S2. Calibration curve of the ABTS method by SIA, using a trolox standard in a range of 10 to 300 µmol L^{-1}.

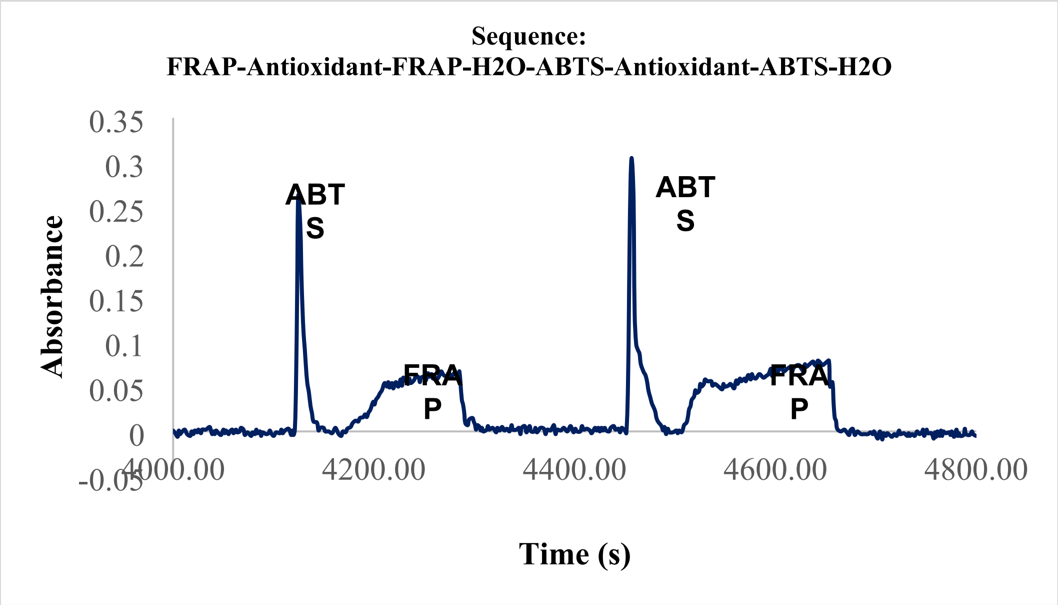

Figure S3. FIAgram of experiment 1 (sequence FRAP–Antioxidant–FRAP–H_2O–ABTS–Antioxidant–ABTS–H_2O) using trolox at 10 µmol L^{-1}.

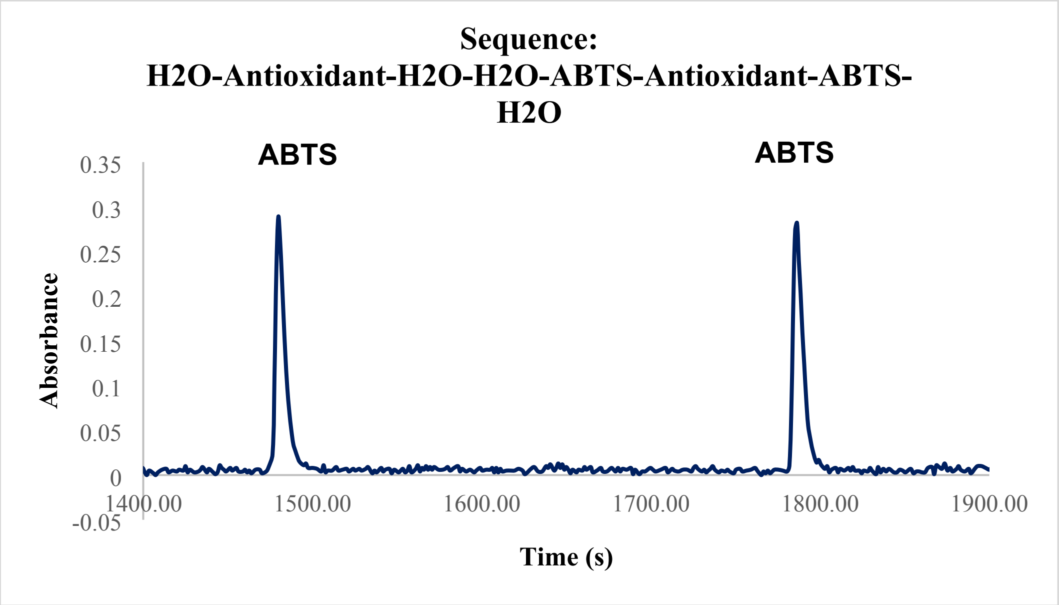

Figure S4. FIAgram of the blank from experiment 1 (sequence H_2O–Antioxidant–H_2O–H_2O–ABTS–Antioxidant–ABTS–H_2O) using trolox at 10 μmol L^{-1}.

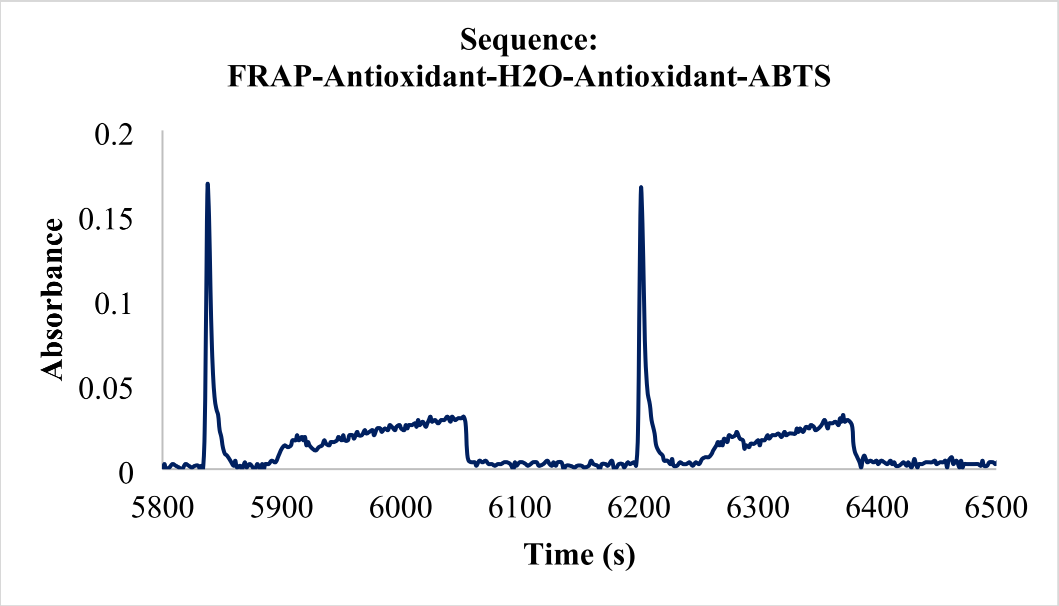

Figure S5. FIAgram of experiment 3 (sequence FRAP–Antioxidant–H_2O–Antioxidant–ABTS) using trolox at 10 μmol L^{-1}.

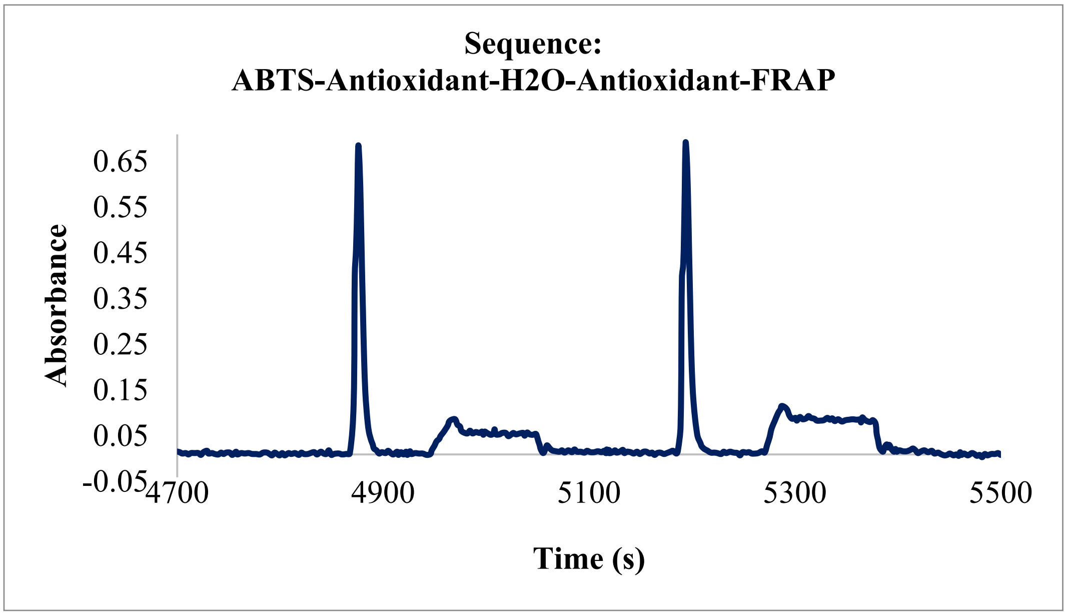

Figure S6. FIAgram of experiment 4 (sequence ABTS–Antioxidant–H_2O–Antioxidant–FRAP) using trolox at 10 µmol L^{-1}.

Table S5. Results for the choice of the aspiration sequence, based on the estimated absorbance for each assay using trolox at 60 µmol L^{-1}. For all sequences after aspirating FRAP and antioxidant this bolus was sending to reactor.

Experiment	Aspiration sequence	A_{FRAP} a 593 nm	A_{ABTS} a 734 nm
1	FRAP–Antioxidant-ABTS-Antioxidant	0.154±0.002	0.126±0.003
2	Antioxidant-FRAP-Antioxidant-ABTS	0.138±0.010	0.110±0.003
3	FRAP–Antioxidant-Antioxidant-ABTS	0.143±0.003	0.108±0.003
4	Antioxidant-FRAP-ABTS-Antioxidant	0.140±0.006	0.114±0.005

Table S6. DCC in 3 blocks with 3 central points, to evaluate the factors FRAP concentration, $ABTS^{\bullet+}$ radical concentration, aspirate volume and flow rate of the FRAP/ABTS-SIA system, using AFRAP and ABTS as response variables and trolox 60 µmol L^{-1} as standard.

Block	Concentration FRAP [a]	Concentration $ABTS^{\bullet+}$ [a]	Aspiration Volumes [b]	Flow rate [c]	A_{FRAP} 593 nm	A_{ABTS} 734 nm
1	100	20	28/23/23/28	25	0.129	0.086
1	100	50	38/33/33/38	25	0.166	0.109
1	80	20	38/33/33/38	25	0.172	0.130
1	100	20	38/33/33/38	35	0.177	0.137
1	90	35	33/28/28/33	30	0.155	0.107
1	80	20	28/23/23/28	35	0.133	0.094
1	100	50	28/23/23/28	35	0.128	0.096
1	90	35	33/28/28/33	30	0.151	0.109
1	90	35	33/28/28/33	30	0.152	0.114
1	80	50	28/23/23/28	25	0.125	0.103
1	80	50	38/33/33/38	35	0.175	0.162
2	100	50	38/33/33/38	35	0.170	0.145
2	80	50	28/23/23/28	35	0.114	0.106
2	100	50	28/23/23/28	25	0.117	0.103
2	80	50	38/33/33/38	25	0.171	0.133
2	80	20	38/33/33/38	35	0.170	0.130
2	90	35	33/28/28/33	30	0.150	0.112
2	90	35	33/28/28/33	30	0.155	0.117
2	90	35	33/28/28/33	30	0.158	0.115
2	100	20	28/23/23/28	35	0.128	0.093
2	80	20	28/23/23/28	25	0.119	0.098
2	100	20	38/33/33/38	25	0.176	0.130
3	90	35	23/18/18/23	30	0.103	0.111
3	90	35	33/28/28/33	30	0.147	0.111
3	90	5	33/28/28/33	30	0.148	0.000
3	90	35	33/28/28/33	30	0.154	0.113
3	90	35	33/28/28/33	30	0.153	0.107
3	90	35	33/28/28/33	40	0.157	0.111
3	90	65	33/28/28/33	30	0.141	0.109
3	90	35	33/28/28/33	20	0.145	0.122
3	90	35	43/38/38/43	30	0.187	0.147
3	70	35	33/28/28/33	30	0.146	0.107
3	110	35	33/28/28/33	30	0.149	0.110

[a] Values expressed in percentages (v/v); [b] volume expressed in µL (Antioxidant/FRAP/ABTS/Antioxidant); [c] flow rate expressed in µL s^{-1}.

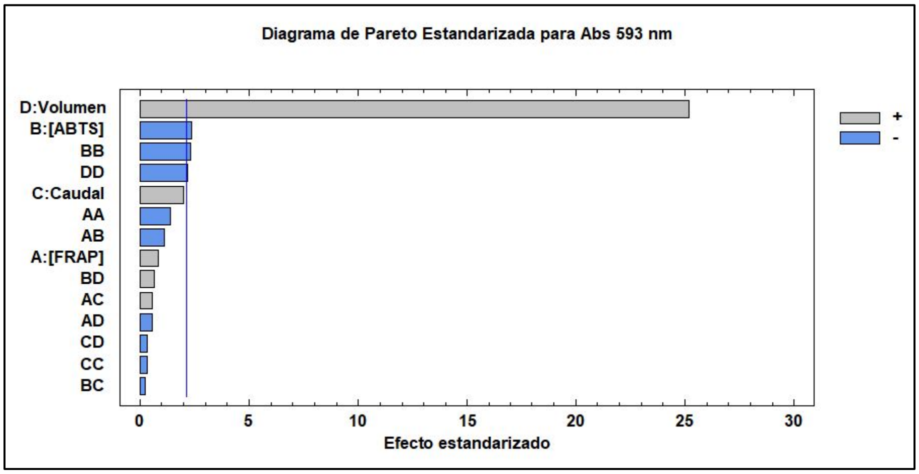

Figure S7. Standardized Pareto chart with binary interactions of the factors, to maximize the response of the FRAP/ABTS-SIA system, using AFRAP as the response variable.

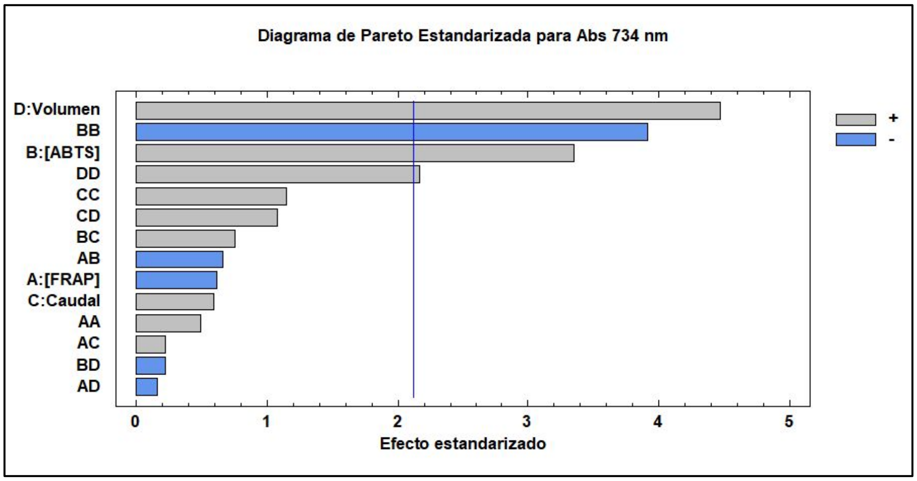

Figure S8. Standardized Pareto chart with binary interactions of the factors, to maximize the response of the FRAP/ABTS-SIA system, using AABTS as the response variable.

Table S7. Experiments to determine optimal concentration values for the FRAP reagent and the $ABTS^{\bullet+}$ radical using trolox at 60 µmol L^{-1}.

Experiment	FRAP Concentration	$ABTS^{\bullet+}$ Concentration	A_{FRAP}	A_{ABTS}
1	96 %	27 %	0.185±0.009	0.167±0.018
**2**	**70 %**	**50 %**	**0.195±0.006**	**0.179±0.002**
3	80 %	40 %	0.197±0.007	0.160±0.005

Experiments set at a flow rate of 40 µL s^{-1} and aspirate volume (Antioxidant (43 µL)-FRAP(38 µL)-ABTS(38 µL)-Antioxidant (43 µL)).

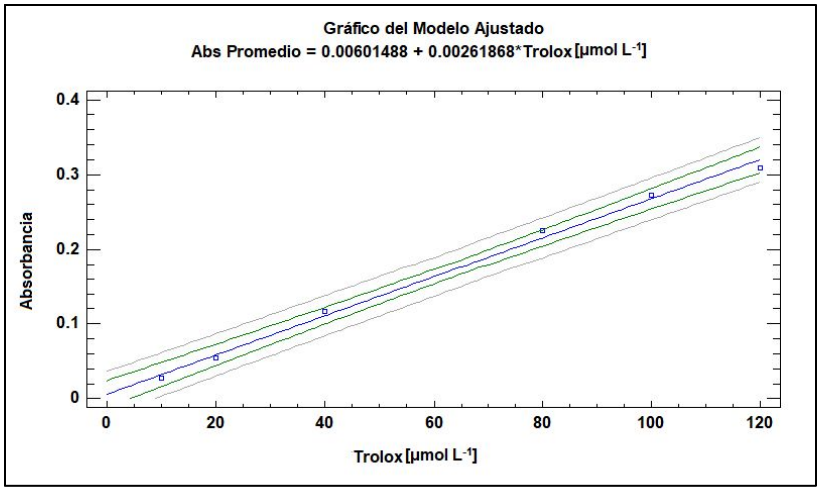

Figure S9. Average curve for antioxidant capacity determination for the FRAP assay using the FRAP/ABTS-SIA system.

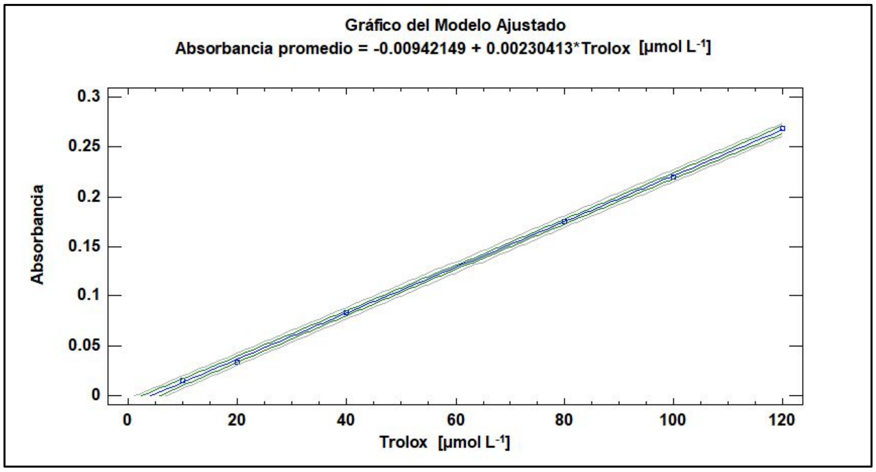

Figure S10. Average curve for antioxidant capacity determination for the ABTS assay using the FRAP/ABTS-SIA system.

Table S8. Results for measuring the precision of antioxidant capacity quantification in Trolox and honey coffee extract for the FRAP assay by FRAP/ABTS-SIA.

	Honey coffee extract 0.5%.			Trolox 60 μmol L^{-1}.		
**Day**	**Replica 1**	**Replica 2**	**Average**	**Replica 1**	**Replica 2**	**Average**
1	50.70	50.05	50.37	59.06	60.49	59.77
2	49.22	49.86	49.54	61.97	62.48	62.22
3	48.88	48.75	48.81	61.28	61.02	61.15
4	47.22	49.25	48.24	59.86	61.41	60.63
5	48.30	49.73	49.01	59.74	59.74	59.74
	**Total average**		49.19			60.70

Table S9. Results for measuring the precision of antioxidant capacity quantification in Trolox and honey coffee extract for the ABTS assay by FRAP/ABTS-SIA.

	**Honey coffee extract 0.5%.**			**Trolox 60 µmol L^{-1}.**		
**Day**	**Replica 1**	**Replica 2**	**Average**	**Replica 1**	**Replica 2**	**Average**
1	62.51	61.35	61.93	59.46	60.04	59.75
2	60.48	61.06	60.77	58.59	59.61	59.10
3	62.36	61.64	62.00	60.77	61.64	61.20
4	63.81	63.52	63.67	57.58	58.30	57.94
5	62.65	62.94	62.80	59.17	59.32	59.25
	**Total average**		62.23			59.45

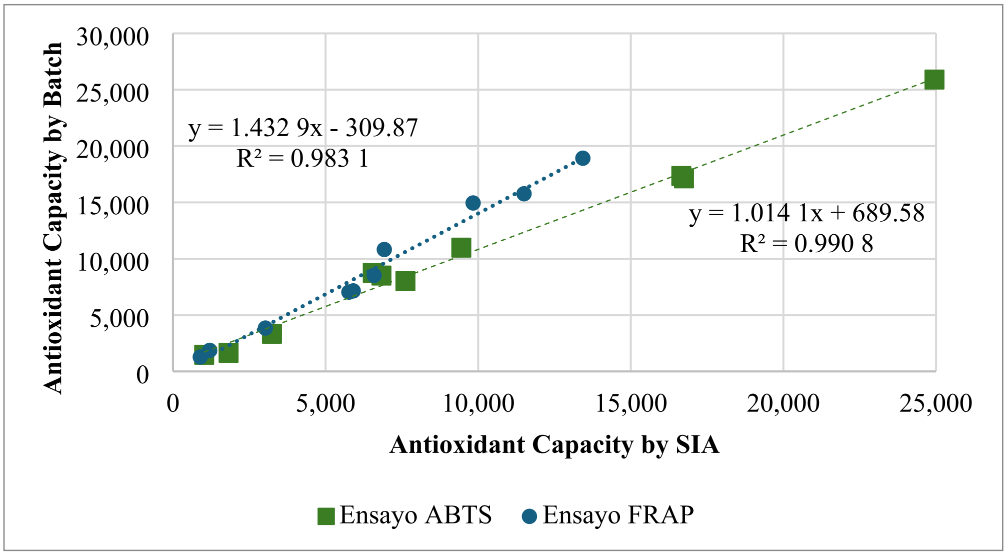

Figure S11. Correlation of antioxidant capacity (AC) of FRAP and ABTS assays, by FRAP/ABTS-SIA and microplate for 10 food samples.

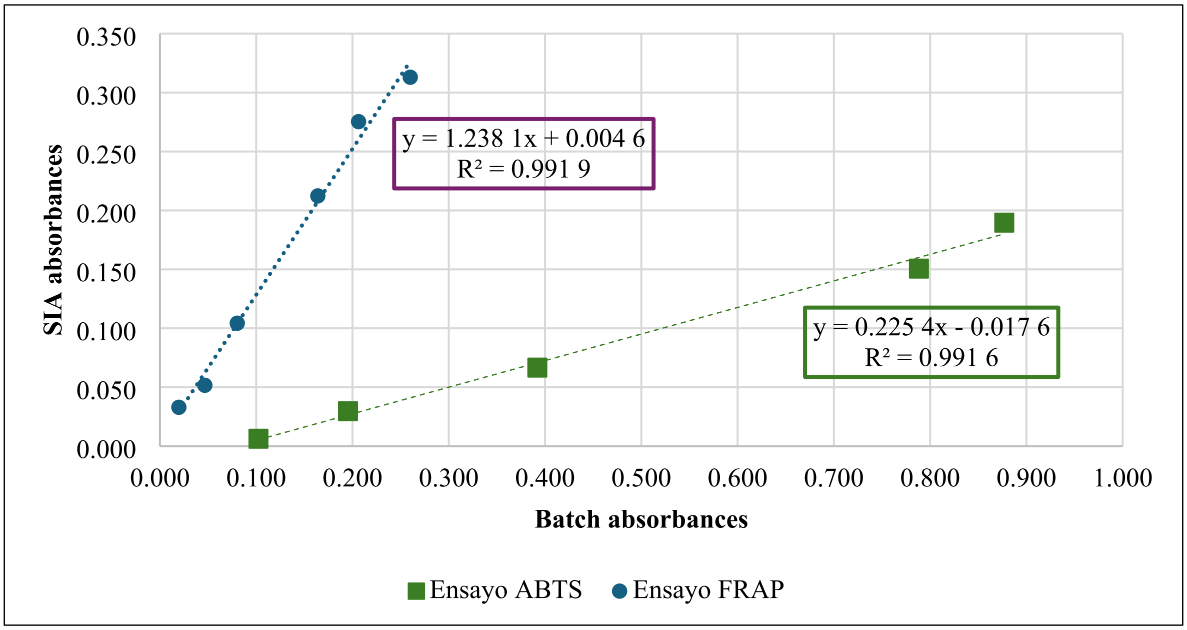

Figure S12. Correlation of absorbances of the FRAP and ABTS assays, by the FRAP/ABTS-SIA system vs microplate when using Trolox concentrations of 10 to 120 µmol L^{-1} for the FRAP assay and 10 to 100 µmol L^{-1} for the ABTS assay.
